# Pace and parity predict the short‐term persistence of small plant populations

**DOI:** 10.1002/ece3.11044

**Published:** 2024-02-20

**Authors:** Michelle DePrenger‐Levin, Michael B. Wunder

**Affiliations:** ^1^ Denver Botanic Gardens Denver Colorado USA; ^2^ Department of Integrative Biology University of Colorado Denver Denver Colorado USA

**Keywords:** COMPADRE plants matrix database, demographic stochasticity, environmental stochasticity, life history strategy, matrix population model

## Abstract

Life history traits are used to predict asymptotic odds of extinction from dynamic conditions. Less is known about how life history traits interact with stochasticity and population structure of finite populations to predict near‐term odds of extinction. Through empirically parameterized matrix population models, we study the impact of life history (reproduction, pace), stochasticity (environmental, demographic), and population history (existing, novel) on the transient population dynamics of finite populations of plant species. Among fast and slow pace and either a uniform or increasing reproductive intensity or short or long reproductive lifespan, slow, semelparous species are at the greatest risk of extinction. Long reproductive lifespans buffer existing populations from extinction while the odds of extinction of novel populations decrease when the reproductive effort is uniformly spread across the reproductive lifespan. Our study highlights the importance of population structure, pace, and two distinct aspects of parity for predicting near‐term odds of extinction.

## Graphical abstract

Through empirically parameterized matrix population models, we study the impact of life history (reproduction, pace), stochasticity (environmental, demographic), and population history (existing, novel) on the near‐term population dynamics of finite populations of plant species. Among fast and slow pace and either a uniform or increasing reproductive intensity or short or long reproductive stage duration, slow, semelparous species are at the greatest risk of extinction. Our study highlights the importance of population structure, pace, and two distinct aspects of parity for predicting near‐term extinction risk.

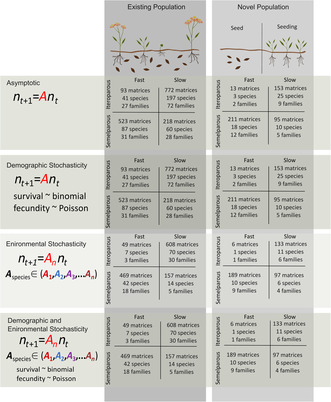

## 1 INTRODUCTION

Changes in climate and land use often lead to reduced population sizes and increased odds of extinction (Terry et al., 2022). Warming climates push distribution ranges to higher latitudes or elevations (IPCC, 2022; Thurman et al., 2020). The ability of a species, whether native or introduced, to invade a novel habitat depends on the species traits, physiological tolerances, and stochastic processes (Iles et al., 2016). Life history theory has been used to predict which species will persist in the long‐term (Schmid et al., 2022; Shaw et al., 2020; Stearns, 1976). Conservation action, however, is focused on short‐term dynamics for populations that are already small or restricted in range. Short‐term, transient dynamics differ from long‐term stable structure dynamics (Ellis & Crone, 2013; Iles et al., 2016; Kendall, 1998; Koons et al., 2016), yet most studies use sensitivity analysis of long‐term growth rate in large populations to study how life history strategies mitigate the effects of demographic stochasticity in the absence of environmental variability (Iles et al., 2016; Jeppsson & Forslund, 2012), or the effects of environmental variation in the absence of demographic stochasticity (Schmid et al., 2022). The minimum population size reached during the transient phase is a good indicator of long‐term population viability, but high fecundity is correlated with both small minimum population sizes during the transient phase and high asymptotic population growth rates (Iles et al., 2016). It is unclear if generalizations can be made by the life history of the odds of extinction due to the combined impacts of finite population size, life history, and stochastic processes in short time periods likely within the transient phase when it is possible to respond with conservation policies and actions.

In large populations, random variation in death or reproductive rates among individuals has a small impact on the long‐term population growth rate (Kokko & Ebenhard, 1996) but in small populations, demographic stochasticity reduces growth rates and increases the probability of extinction. Pace‐of‐life (hereafter, “pace”) can mitigate these risks of extinction. So‐called “Fast” species are associated with short generation times, many offspring per reproductive event, fast population turnover rates, demographic lability, the ability to evolve quickly, and a greater risk of short‐term population fluctuation (Le Coeur et al., 2022; Parry, 1981; Schmid et al., 2022). By contrast, so‐called “Slow” species have longer generation times, higher age of reproductive maturity, few offspring per reproductive event, reduced temporal variation vital rates in response to environmental variation, and slower population turnover that leads to greater near‐term persistence (Le Coeur et al., 2022; Schmid et al., 2022; Stearns, 1976). Conservation action considers near‐term outcomes, when the persistence of fast‐paced populations should depend on the initial size of the population and slow populations should be more robust to demographic and environmental stochasticity regardless of population size through demographic buffering (Le Coeur et al., 2022).

Although pace is often used as a proxy for the effects of life history strategy on demographics, it interacts with parity to influence the odds of extinction. Parity can be considered in terms of the duration of the reproductive lifespan (mature life expectancy in Salguero‐Gómez, 2017), or the shape of reproductive intensity over the reproductive lifespan, hereafter referred to as ‘shape’ (Baudisch & Stott, 2019; Demetrius, 1978). Plants with long mature life expectancies and uniform reproductive intensity across age (iteroparity) tend to experience increasing population growth rates, whereas species with short reproductive lifespan and variable intensity across age (semelparity) tend to experience population declines more frequently (Salguero‐Gómez, 2017). Species respond to environmental variability not just in terms of duration or intensity of seed production, but also seed germination rate. Iteroparous species mitigate environmental stochasticity by producing large seeds, maximizing seedling survival but reducing the likelihood of contributing to the soil seed bank (Crawley, 1996; Honda, 2008). Semelparous life histories mitigate environmental stochasticity by producing many small seeds that persistent as a soil seed bank (Guo et al., 2022; Rosbakh et al., 2022), and thereby delay germination in response to environmental variability (Saatkamp et al., 2014). Thus, iteroparous life histories reduce impacts of environmental stochasticity by prolonging seed production whereas semelparous life histories bet hedge by prolonging seed germination. Large propagules with high‐survival rates reduce demographic stochasticity while a larger number of propagules reduces the impact of environmental stochasticity (Simberloff, 2009).

Here, we explored how life history strategies interact with finite population size, stochastic processes, and stage distribution to influence the probability of near‐term extinction. First, we explored the extent to which plant life history traits that have evolved in response to a variable environment can predict transient population dynamics. We then quantified the comparative influences of initial population size, demographic stochasticity, and environmental stochasticity on population dynamics for each life history strategy. Second, to extend the model to species that lack demographic data, we quantified the association between a continuous measure of reproductive intensity and parity (iteroparity or semelparity) and then quantified the effects of categorized pace, parity, stochastic processes, and initial size of populations founded as either the seed or seedling stage on the odds of extinction.

## 2 METHODS

We used simulations to connect life history theory with the prediction of short‐term persistence for small finite existing populations of rare and threatened species, and newly introduced populations that are not at equilibrium (stable stage distribution) as is the case for managed restoration and reintroductions or for dispersal events. We first compared the impact of population size on long‐term, asymptotic growth dynamics with the impacts of demographic and environmental stochasticity. Then, we quantified the relative effects of categorized pace (‘fast’ or ‘slow’), population size, stochastic processes, and either a continuous measure of reproductive intensity (‘shape’) or a categorical measure of reproductive lifespan (‘parity’) on the differences in short‐term odds of extinction. Lastly, because managed populations are initiated from seed or from planting vegetative individuals, we simulated the impacts of unstable stage structure, initial population size, and stochastic processes on the odds of near‐term extinction by life history.

### 2.1 Empirical data

We gathered empirically derived stage‐based population models from the COMPADRE Plant Matrix Database v6.22.5.0 (created 2022‐05‐11; Salguero‐Gomez et al., 2015) that (1) were ergodic and irreducible, (2) were modeled on an annual time step (Iles et al., 2016), and (3) did not explicitly parse clonal growth into a separate matrix. This subset resulted in 1606 matrices representing multiple years and/or populations of 317 plant species. Multiple matrices for the same species were considered individually for analyses of asymptotic growth rates and demographic stochasticity but were considered collectively to analyze the effects of environmental stochasticity. We addressed the possibility that patterns attributed to life history are confounded with nonindependence from phylogenetic relatedness among species (Appendix S1). However, due to the lack of a robust phylogeny that covers all species used in this analysis, and because life history was derived from individual matrices and not tied to each species, we did not use a phylogenetic covariance matrix in our analyses.

### 2.2 Defining life histories

The tradeoff between a life history defined by rapid growth with high fecundity (fast pace of life) and another by slow growth with low fecundity (slow pace) is insufficient for describing life history variation among plants (Salguero‐Gomez et al., 2016). To predict population dynamics, we therefore modeled plant life history strategies using continuous measures of reproduction and survival (c.f. Salguero‐Gomez et al., 2016). However, to apply predictions to species for which we do not have detailed demographic data, we need recognizable categories of pace and parity. While stage and size are better predictors of demographic behavior than age in plants, age‐specific senescence defines plant life history (Picó & Retana, 2008).

We used the age of reproductive maturity to quantify the effects of the pace of life on the odds of extinction (Table 1). The age of reproductive maturity measures the lifespan and time needed for a population to recruit offspring and become self‐sustaining (Albrecht et al., 2019; Cochran & Ellner, 1992). Recruitment can be expected to be observed within a reasonable length of time for fast life histories but may take longer than a typical study for slow life histories. We estimated the age of reproductive maturity using the Markov chain approach in the R package *Rage* (Jones et al., 2022) and specifying the beginning of life as the first non‐dormant stage. Age is then the estimated number of years after germination, similar to using annual ring production to age plants (Perkins et al., 2006). Fast species were defined as reaching reproductive maturity in fewer than 3 years. For semelparous species where reproduction is followed by death, fast species are annuals and biennials whereas slow species are long‐lived perennials. For iteroparous species which reproduce annually for several years after reaching reproductive maturity, fast species are reproductive for approximately half of their lifespans whereas long‐lived species can be reproductive for most of their lifespan (Appendix S1, Figure S5). The threshold of 3 years was selected to create a fairly even sample size among life history categories while reflecting traits that were observable without detailed demographic data.

**TABLE 1:** Parameter definitions and priors for the main effects in the models of the odds of extinction.

Parameter		Covariates	Prior	Equation 4a	Equation 4b	Equation 5a	Equation 5b
Intercept	$\beta_{0}$		$\times \text{Normal}\left(\beta_{0}|\mu ,\sigma^{2}\right)\times \text{Normal}\left(\mu |0,0.0001\right)\times \text{Normal}\left(\sigma^{2}|0,100\right)$	x	x	x	x
**Main effects**							
Pace	$\beta_{1}$ $\beta_{2}$	$x_{1}=\left\{\begin{array}{c} \text{Fast}\\ \;\text{Slow} \end{array}\right.$	$\times \text{Normal}\left(\beta |0,0.01\right)$	x	x	x	x
Shape of reproduction (Shape)	$\beta_{3}$	$x_{2}$	$\times \text{Uniform}\left(\beta |-3,3\right)$	x		x	
Parity	$\beta_{4}$ $\beta_{5}$	$x_{3}=\left\{\begin{array}{c} \text{Iteroparous}\\ \text{Semelparous} \end{array}\right.$	$\times \text{Normal}\left(\beta |0,0.01\right)$		x		x
Treatment	$\beta_{6}$ $\beta_{7}$ $\beta_{8}$ $\beta_{9}$	$x_{4}=\left\{\begin{array}{c} \text{Asymptotic}\\ \text{Demographic}\\ \text{Environmental}\\ \text{Demographic}+\text{Envrionmental} \end{array}\right.$	$\times \text{Normal}\left(\beta |0,0.01\right)$	x	x	x	x
Founding population size (PopSz)	$\beta_{10}$	$x_{5}=\left[1,10,100,500,1000\right]$	$\times \text{Uniform}\left(\beta |-3,3\right)$	x	x	x	x
Founding stage (Stage)	$\beta_{11}$ $\beta_{12}$	$x_{6}=\left\{\begin{array}{c} \text{Seed}\\ \text{Seedling} \end{array}\right.$	$\times \text{Normal}\left(\beta |0,0.01\right)$			x	x
**Interactions**							
Pace × Parity	$\beta_{13}$ $\beta_{14}$ $\beta_{15}$ $\beta_{16}$	$x_{1}x_{3}=\left\{\begin{array}{c} \;\text{Fast Iteroparous}\;\\ \text{Fast Semelparous}\\ \text{Slow Iteroparous}\\ \text{Slow Semelparous} \end{array}\right.$	$\times \text{Normal}\left(\beta |0,0.01\right)$		x		x
Pace × Shape	$\beta_{17}$ $\beta_{18}$	$x_{1}x_{2}=\left\{\begin{array}{c} \text{Fast}\times \text{Shape}\\ \text{Slow}\times \text{Shape} \end{array}\right.$	$\times \text{Normal}\left(\beta |0,0.01\right)$	x		x	
Pace × PopSz	$\beta_{19}$ $\beta_{20}$	$x_{1}x_{5}=\left\{\begin{array}{c} \text{Fast}\times \text{Population Size}\\ \text{Slow}\times \text{Population Size} \end{array}\right.$	$\times \text{Normal}\left(\beta |0,0.01\right)$	x	x	x	x
Pace × Treatment	$\beta_{21}$ $\beta_{22}$ $\beta_{23}$ $\beta_{24}$ $\beta_{25}$ $\beta_{26}$ $\beta_{27}$ $\beta_{28}$	$x_{4}x_{1}=\left\{\begin{array}{c} \;\text{Asymptotic Fast}\;\\ \text{Asymptotic Slow}\\ \text{Demographic Fast}\\ \text{Demographic Slow}\\ \text{Environmental Fast}\\ \text{Environmental Slow}\\ \text{Demo}+\text{Environ Fast}\\ \text{Demo}+\text{Environ Slow} \end{array}\right.$	$\times \text{Normal}\left(\beta |0,0.01\right)$	x	x	x	x
Parity × PopSz	$\beta_{29}$ $\beta_{30}$	$x_{3}x_{5}=\left\{\begin{array}{c} \text{Iteroparous}\times \text{PopSz}\\ \text{Semelparous}\times \text{PopSz} \end{array}\right.$	$\times \text{Normal}\left(\beta |0,0.01\right)$		x		x
Shape × PopSz	$\beta_{31}$	$x_{3}x_{5}$	$\times \text{Uniform}\left(\beta |-5,5\right)$	x		x	
Parity × Treatment	$\beta_{32}$ $\beta_{33}$ $\beta_{34}$ $\beta_{35}$ $\beta_{36}$ $\beta_{37}$ $\beta_{38}$ $\beta_{39}$	$x_{4}x_{3}=\left\{\begin{array}{c} \;\text{Asymptotic Iteroparous}\;\\ \text{Asymptotic Semelparous}\\ \text{Demographic Iteroparous}\;\\ \text{Demographic Semelparous}\\ \text{Environmental Iteroparous}\;\\ \text{Environmental Semelparous}\\ \text{Demo}+\text{Environ Iteroparous}\;\\ \text{Demo}+\text{Environ Semelparous} \end{array}\right.$	$\times \text{Normal}\left(\beta |0,0.01\right)$		x		x
Treatment × PopSz	$\beta_{40}$ $\beta_{41}$ $\beta_{42}$ $\beta_{43}$	$x_{4}x_{5}=\left\{\begin{array}{c} \text{Asymptotic}\times \text{PopSz}\\ \text{Demographic}\times \text{PopSz}\\ \text{Environmental}\times \text{PopSz}\\ \text{Demo}+\text{Envrion}\times \text{PopSz} \end{array}\right.$	$\times \text{Normal}\left(\beta |0,0.01\right)$	x	x	x	x
Pace × Stage	$\beta_{44}$ $\beta_{45}$ $\beta_{46}$ $\beta_{47}$	$x_{6}x_{1}=\left\{\begin{array}{c} \;\text{Seed}\times \text{Fast}\;\\ \text{Seed}\times \text{Slow}\\ \text{Seedling}\times \text{Fast}\\ \text{Seedling}\times \text{Slow} \end{array}\right.$	$\times \text{Normal}\left(\beta |0,0.01\right)$			x	x
Parity × Stage	$\beta_{48}$ $\beta_{49}$ $\beta_{50}$ $\beta_{51}$	$x_{6}x_{3}=\left\{\begin{array}{c} \;\text{Seed}\times \text{Iteroparous}\;\\ \text{Seed}\times \text{Semelparous}\\ \text{Seedling}\times \text{Iteroparous}\;\\ \text{Seedling}\times \text{Semelparous} \end{array}\right.$	$\times \text{Normal}\left(\beta |0,0.01\right)$			x	x
PopSz × Stage	$\beta_{52}$ $\beta_{53}$	$x_{6}x_{5}=\left\{\begin{array}{c} \text{Seed}\times \text{PopSz}\\ \text{Seedling}\times \text{PopSz} \end{array}\right.$	$\times \text{Normal}\left(\beta |0,0.01\right)$			x	x
Treatment × Stage	$\beta_{54}$ $\beta_{55}$ $\beta_{56}$ $\beta_{57}$ $\beta_{58}$ $\beta_{59}$ $\beta_{60}$ $\beta_{61}$	$x_{4}x_{6}=\left\{\begin{array}{c} \;\text{Asymptotic Seed}\\ \text{Asymptotic Seedling}\\ \text{Demographic Seed}\\ \text{Demographic Seedling}\\ \text{Environmental Seed}\\ \text{Environmental Seedling}\\ \text{Demo}+\text{Environ Seed}\\ \text{Demo}+\text{Environ Seedling} \end{array}\right.$	$\times \text{Normal}\left(\beta |0,0.01\right)$			x	x

*Note*: All categorical variables are centered $\beta_{i}=\beta_{i}-\bar{\beta_{i}}$, continuous variables are centered and scaled.

To understand the effects of reproductive intensity and duration on the dynamics of established populations, we quantified variation in the intensity of reproduction across ages of reproductive maturity ($\alpha_{\text{adultlong}}$, reproductive lifespan; Table 1) and then compared this to the categorical short (‘semelparous’) and long (‘iteroparous’) duration of reproduction. As a continuous measure of reproductive intensity, we used shape (𝑆 in Baudisch & Stott, 2019) and compared the variation in demographic dynamics within and among discrete life history categories to determine how well these generalizations describe the response to demographic and environmental stochasticity (Appendix S1). Senescence results in an increase in mortality or a decrease in fertility with age (Caswell & Salguero‐Gomez, 2013). However, aging does not always result in senescence (Baudisch & Stott, 2019). Fertility can be unchanged with age (negligible senescence) or even increase with age (negative senescence) and these aging patterns have evolved independently of lifespan (Baudisch & Stott, 2019). The shape of reproduction was measured as the cumulative reproductive output from the first age of reproduction $m\left(\alpha \right)$ to the last $m\left(\beta \right)$. The rate of reproduction B at age α is zero. The function assumes the age of reproductive cessation is the age at last reproduction plus the difference between the final two reproductive ages to calculate shape as:

(1) $$S=\frac{1}{\mathrm{\tau B}}\left(\sum_{x=\alpha }^{\beta }B\left(x\right)-\frac{\mathrm{\tau B}}{2}\right)$$

Where the reproductive lifespan τ is used to factor out pace P and make shape comparable across the diverse life histories of plants. For instance, for an annual with ages [0, 1], the first age of reproduction is α=1. The Markov chain approach in the R package *Rage* assumes the life table is incomplete and adds age 2 for ages [0, 1, 2] where reproduction ceases at age 2 (β=2 and $B\left(1\right)=0$) and Equation 1 is applied to the interval from age 1 to 2.

Shape approaching 0.5 represents positive senescence where reproduction is concentrated early in life and diminishes with age. Only two species, each represented by a single matrix, had this shape, and so were excluded from simulations (*Tillandsia deppeana* Steud. S=0.30 and *Eupatorium resinosum* Torr. ex DC. S=0.39). S approaches 0 for plants with minimal growth after reaching the age of reproductive maturity and constant intensity of reproductive output thereafter (Purchase et al., 2022). Shape approaching −0.5 represents negative senescence with reproduction concentrated at the end of life where size and reproductive output both continually increase after reaching the age of reproductive maturity. Shape describes how reproductive intensity varies over the reproductive lifespan regardless of the duration of reproductive longevity. For categorical classification, an ‘iteroparous’ reproductive strategy was defined as three or more years in the reproduction stage (reproductive longevity) whereas a ‘semelparous’ strategy was defined as fewer than 3 years of reproductive longevity. Reproductive longevity was estimated using the R code adapted by COMPADRE from Caswell (2001) as the mean life expectancy if entering the life cycle at the age of reproductive maturity. Categorizing matrices in this manner yielded 93 fast iteroparous matrices (41 species, 27 families), 523 fast semelparous matrices (87 species, 31 families), 772 slow iteroparous matrices (197 species, 72 families), and 218 slow semelparous matrices (60 species, 28 families).

### 2.3 Simulations

**FIGURE 1:**
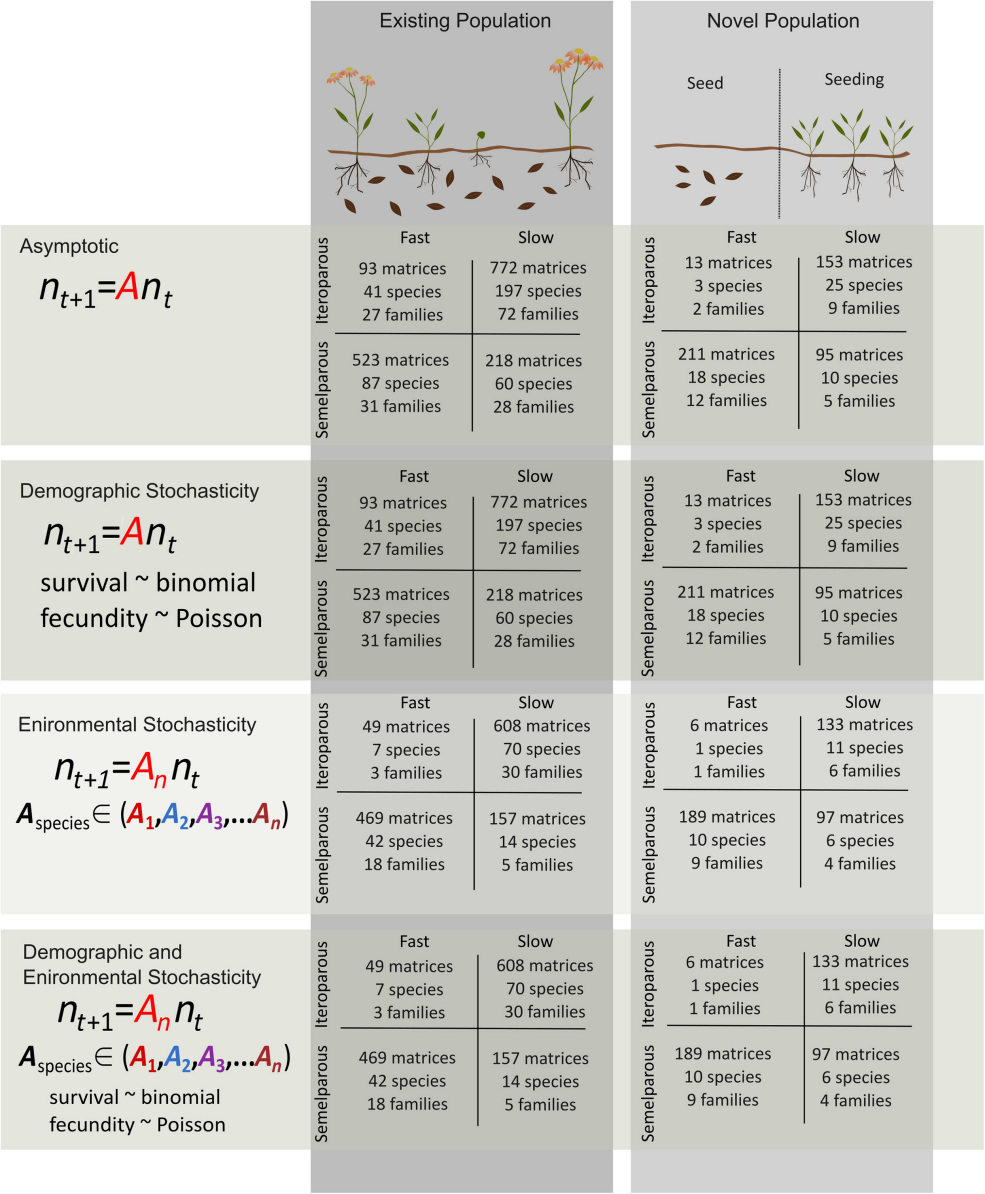
Study design includes four experimental treatments (asymptotic, demographic stochasticity, environmental stochasticity, demographic and environmental stochasticity) for two population histories (existing, novel) to test the effect of life history (fast iteroparous, fast semelparous, slow iteroparous, slow semelparous) and population size (1, 10, 50, 100, 1000) on the odds of extinction. Sample sizes are listed for matrices, species, and families.

We projected simulated population dynamics for each of the four life history categories (fast iteroparous, fast semelparous, slow iteroparous, slow semelparous) for each of two different population histories (existing population, newly introduced population) under four stochastic conditions (asymptotic in the absence of stochasticity, demographic stochasticity, environmental stochasticity, demographic and environmental stochasticity; Figure 1). We used the simulated outcomes for existing populations to model the effects of life history, population size, demographic stochasticity, and environmental stochasticity on the mean odds of extinction. We used the simulated outcomes for newly introduced populations to model the effects of life history, population size, initial population stage (seed or vegetative), demographic stochasticity, and environmental stochasticity on the mean odds of extinction. For both population histories, we compared models that included categorical pace and reproductive lifespan to models that included categorical pace and continuous shape of reproduction. We explored the distribution of demographic rates among life history categories for empirical matrix population models that included a seed stage. We found that demographic rates including germination, survival, and growth met expectations by life history category (Appendix S1, Figure S5).

To assess the effects of initial population size on population persistence for existing populations, simulations were initialized with one of five abundances (1, 10, 100, 500, or 1000) distributed across stages proportional to the stable stage distribution. To quantify the effect of the founding stage on the dynamics of newly introduced populations, we used matrix models that explicitly included a seed stage, resulting in 472 matrices from 56 species in 28 families (Figure 1). For simulations of newly introduced populations, initial abundances were assigned to either the ‘seed’ or smallest, non‐dormant ‘seedling’ stage. When matrices had multiple seed stages, the initial population size was assigned to the class with the largest abundance among the seed stages at stable distribution (Iles et al., 2016).

The initial simulation for each life history category and population history was designed to model the expected asymptotic behavior using time‐invariant parameters (Equation 2).

(2) $$n_{t}=An_{t-1}$$

where A is a matrix population model and $n_{t}$ represents the vector of stage‐specific abundances at time t.

Demographic stochasticity was modeled by drawing the stage‐specific survival and transitions from a binomial distribution and the number of offspring (fecundity) from a Poisson distribution with stage‐specific expectations from the A matrix (Figure 1).

Environmental stochasticity was modeled for species with three or more years and/or sites of demographic information of the same matrix dimensions and life history category (Figure 1). Therefore, these simulations include both environmental stochasticity and spatial variation in life history. However, spatial variation was reduced by grouping matrices within a species that fell into the same life history category. The original matrix dimensions were maintained to reduce bias in the inferred demographic parameters (Salguero‐Gomez & Plotkin, 2010). Environmental stochasticity was simulated with time‐variant models where a matrix was selected at random for each annual transition assuming the environmental condition in the current year is independent of the conditions in the previous year (Equation 3).

(3) $$n_{t}=A_{t}n_{t-1}$$

where $A_{t}$ is randomly selected from the set of matrix population models for each annual t transition and $n_{t}$ represents the vector of stage abundances at time t. Note that eight species contained matrixes classified into different life history categories (Appendix S1). Populations respond to local conditions through changes in the timing or duration of vital rates (reproductive maturity, reproductive longevity) and can maintain high demographic variability among individuals despite ecological and evolutionary selective pressures (Steiner et al., 2021). The stable stage distribution was calculated from the resulting mean matrix of averaged demographic parameters across matrices within species for each life history.

The final simulations combined demographic random processes around stage‐specific matrix expectations and environmental stochasticity (Equation 2) among matrices (Figure 1).

We simulated 10,000 projections, sampling matrices or species (by life history) with replacement to proportionally select among life histories for each of the four treatments and five initial population sizes resulting in a sample size of 200,000 (Table 1). Each simulation was projected for 100 years to compare long‐ to near‐term (10 years) odds of extinction. The selected near‐term projection length is relevant to the time in which is it possible to gather empirical data and make conservation decisions but may not capture the full extent of transient dynamics (Stott et al., 2011). The length of the transient period will differ by life history (Stott et al., 2011). The odds of extinction were calculated as the ratio of simulations that projected extinction ($N_{t}=0$) within a specific timeframe to those that did not project extinction. We modeled the mean odds of extinction in a Bayesian framework using JAGS (Plummer, 2003) in R (R Core Team, 2022) as a function of starting population size, pace, shape (Equation 4a) or parity (Equation 4b), stochastic process, and their interactions on scaled and centered N population size and S shape (https://github.com/DenverBotanicGardens/TraitsExtinctionRisk). We explored potential nonindependence among life history traits (Chamberlain et al., 2012, Appendix S1).

(4a) $$\left[\phi ,\beta ,\mu ,\sigma^{2}|y_{i},x\right]\propto \prod_{i=1}^{200,000}\text{binomial}\left(y_{i}|\phi \right)$$

(4b) $$\text{logit}\left(\phi \right)=\beta_{0}+\beta_{1}x_{1}+\ldots +\beta_{43}x_{4}x_{5}$$

To explore the odds of extinction for novel populations, we added the founding propagule stage to compare the effects of shape (Equation 5a) and parity (Equation 5b). We simulated 10,000 projections for each of the four treatments, five initial population sizes, and two founding stages for a sample size of 400,000 (Table 1).

(5a) $$\left[\phi ,\beta ,\mu ,\sigma^{2}|y_{i}\right]\propto \prod_{n}^{400,000}\text{binomial}\left(y_{i}|\phi \right)$$

(5b) $$\text{logit}\left(\phi \right)=\beta_{0}+\beta_{1}x_{1}+\ldots +\beta_{61}x_{4}x_{6}$$

Non‐informative priors, $\text{Normal}\left(0,0.01\right)$, were used for all centered categorical parameters, and $\text{Uniform}\left(-3,3\right)$ for shape and population size. Shape and population size were scaled and centered. We used Markov Chain Monte Carlo (MCMC) to produce posterior distributions of parameter estimates. We ran three chains with 10,000 iterations and a burn‐in period of 5000 iterations, retaining every sixth iteration. We used the Gelman–Rubin diagnostic (R‐hat) and visual inspection to assess the convergence of MCMC chains.

## 3 RESULTS

### 3.1 Simulations of existing populations

**FIGURE 2:**
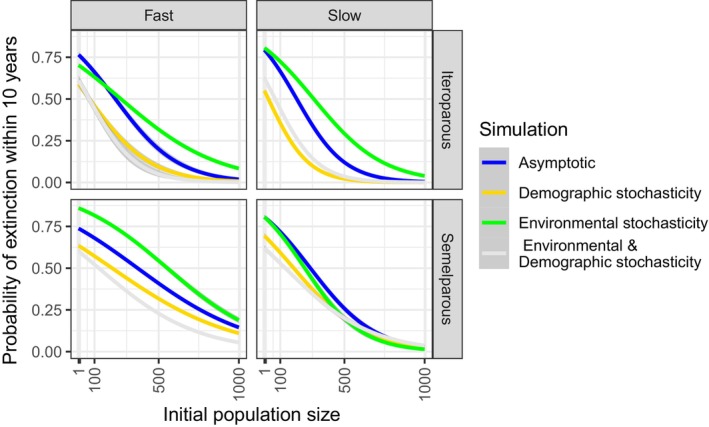
Variation in the probability of extinction within 10 years for populations of initial stable structure as explained by initial population size for life history categories. Extinction is defined as a population dropping below one individual within the projection length ($N_{10}=0$). Life histories are split by pace: fast (age of reproductive maturity <3 years) and slow (age of reproductive maturity ≥3 years), and parity: semelparous (reproductive lifespan <3 years) and iteroparous (reproductive lifespan ≥3 years). Line colors represent simulations of asymptotic conditions (blue), random demographic variation (yellow), environmental variation (green), and both stochastic processes (white) with shaded standard error. A total of 199,760 projections could be classified by pace and parity: *n* = 19,762 Fast Iteroparous, *n* = 50,652 Fast Semelparous, *n* = 106,741 Slow Iteroparous, and *n* = 22,605 Slow Semelparous. The standard error ($\sigma /\sqrt{n}$) is shown trivial in most cases.

As expected, the effects of each parameter on the odds of extinction for existing populations were similar over short‐ (10 year) and long‐term (100 year) projection durations but the magnitude of effects of life history, initial population size, and stochasticity differed and the direction of effect of environmental stochasticity on increasing the initial population size differed between the two. In the near‐term, increasing the initial population size had less of an impact on reducing the odds of extinction while the reverse was true over the long‐term (Appendix S1). In the near‐term, duration of reproduction (parity, Equation 4b) explained variance in odds of extinction across populations size, pace, and stochasticity more effectively than did reproductive intensity (shape, Equation 4a; evidence ratio = $2.53e^{156}$; Figure 2). Neither pace nor parity alone had non‐zero impacts on the odds of extinction (Table 2a). An increase of one standard deviation (SD) of the initial population size (approximately 385 individuals) resulted in a reduction in the odds of extinction of 0.159 (95% HPDI 0.156–0.162). There was a non‐zero effect of treatment on the impact of increasing initial population size. The effect of an SD increase in initial population size was a 79.3% drop in the odds of extinction for environmental and demographic stochasticity, a 39.0% drop for asymptotic conditions, a 38.1% drop for demographic stochasticity alone, and only a 12.0% drop for environmental stochasticity alone (Figure 2). Parity and pace had non‐zero effects on the impact of an SD increase in initial population size (Table 2a). For each increase in one SD of initial population size, the odds of extinction dropped by 33.8% for semelparous life histories and by 57.5% for iteroparous when all other parameters were held at their mean. Under environmental and demographic stochasticity, the odds of extinction dropped by 74.2% for semelparous and 83.4% for iteroparous life histories for each increase of one SD in initial population size. The odds of extinction dropped by 24.9% for fast life histories and 63.9% for slow life histories with every increase of one SD in the initial starting population size when all parameters were held at their mean but by 70.7% for fast and 85.9% for slow under environmental and demographic stochasticity.

**TABLE 2:** Log‐odds of extinction within 10 simulated years for existing populations initiated at stable stage distriubtion for initial population sizes of [1, 10, 100, 500, or 1000] as explained by pace, initial population size, simulated stochasticity, and (a) parity (semelparous: <3 years of reproductive lifespan; iteroparity: ≥3 years of reproductive lifespan) or (b) the shape of reproduction (shape (Baudisch & Stott, 2019) ranges from negative senescence, $\text{Shap}e_{\min}=-0.375$, to negligible senescence, Shape = 0, and towards positive senescence, ${\text{Shape}}_{\max}=0.14$), and their interactions. Parameter estimates, mean, lower and upper highest posterior density interval and standard deviation, are presented on the logit scale and represent the difference from the mean of each parameter. Initial population size and shape of reproduction were scaled and centered. Model fit using JAGS, 3 chains each with 10,000 iterations (first 5000 discarded), thinning to every sixth step. Non‐zero effects are highlighted.

(a) Categorical parity–duration	Mean	SD	Lower HPDI 2.50%	Upper HPDI 97.50%	(b) Continuous shape–intensity	Mean	SD	Lower HPDI 2.50%	Upper HPDI 97.50%
DIC: 191,277.8					DIC: 191,998.1				
(Intercept)	−0.63	0.01	−0.65	−0.62	(Intercept)	−0.45	0.01	−0.47	−0.44
Pace [Fast]	0.08	4.68	−9.04	9.34	Pace [Fast]	0.44	3.14	−5.81	6.78
Pace [Slow]	−0.08	4.68	−9.34	9.04	Pace [Slow]	−0.44	3.14	−6.78	5.81
Parity [Iteroparous]	−0.05	4.64	−9.47	9.05	Shape of Reproduction	−0.20	0.01	−0.21	−0.18
Parity [Semelparous]	0.05	4.64	−9.05	9.47					
Initial population size	−1.84	0.01	−1.86	−1.82	Initial population size	−1.79	0.01	−1.81	−1.77
Asymptotic	0.13	6.15	−12.05	12.52	Asymptotic	0.35	5.00	−9.29	10.23
Demographic Stochasticity	−0.15	6.25	−12.14	12.07	Demographic Stochasticity	−0.19	5.04	−9.97	9.33
Environmental Stochasticity	0.35	6.15	−11.43	12.24	Environmental Stochasticity	0.25	5.05	−9.47	9.72
Environmental and Demographic Stochasticity	−0.34	6.07	−12.33	11.76	Environmental and Demographic Stochasticity	−0.42	4.95	−10.19	9.01
Pace [Fast] × Parity [Iteroparous]	−0.09	5.95	−12.16	11.76	Shape of Reproduction × Pace [Fast]	−0.23	0.01	−0.25	−0.21
Pace [Fast] × Parity [Semelparous]	0.19	5.95	−11.63	11.96					
Pace [Slow] × Parity [Iteroparous]	0.19	5.95	−11.63	11.96	Shape of Reproduction × Pace [Slow]	0.23	0.01	0.21	0.25
Pace [Slow] × Parity [Semelparous]	0.18	5.95	−11.66	12.22					
Pace [Fast] × Initial population size	0.27	0.01	0.25	0.30	Pace [Fast] × Initial population size	0.43	0.01	0.41	0.45
Pace [Slow] × Initial population size	−0.27	0.01	−0.30	−0.25	Pace [Slow] × Initial population size	−0.43	0.01	−0.45	−0.41
Asymptotic × Pace [Fast]	0.07	6.24	−12.19	12.45	Asymptotic × Pace [Fast]	−0.03	5.77	−11.51	11.27
Asymptotic × Pace [Slow]	0.17	6.17	−11.78	12.18	Asymptotic × Pace [Slow]	0.03	6.04	−11.72	11.84
Demographic × Pace [Fast]	−0.08	6.25	−12.79	11.95	Demographic × Pace [Fast]	−0.10	5.98	−11.80	11.54
Demographic × Pace [Slow]	−0.33	6.14	−12.37	11.87	Demographic × Pace [Slow]	−0.43	5.90	−11.87	11.62
Environmental × Pace [Fast]	0.04	6.26	−12.40	11.87	Environmental × Pace [Fast]	0.43	6.01	−11.22	12.14
Environmental × Pace [Slow]	0.24	6.29	−11.74	12.87	Environmental × Pace [Slow]	0.42	5.87	−11.18	11.64
Environ & Demo × Pace [Fast]	−0.05	6.21	−12.15	11.89	Environ & Demo × Pace [Fast]	−0.03	5.89	−11.38	11.67
Environ & Demo × Pace [Slow]	−0.05	6.36	−12.35	12.78	Environ & Demo × Pace [Slow]	−0.30	5.84	−11.39	11.03
Initial population size × Parity [Iteroparous]	−0.33	0.01	−0.35	−0.31	Initial population size × Shape of Reproduction	0.06	0.01	0.04	0.08
Initial population size × Parity [Semelparous]	0.33	0.01	0.31	0.35					
Asymptotic × Parity [Iteroparous]	0.06	6.23	−11.86	12.98	Asymptotic × Shape of Reproduction	0.01	0.01	−0.02	0.03
Asymptotic × Parity [Semelparous]	0.20	6.33	−12.50	12.52					
Demographic × Parity [Iteroparous]	−0.33	6.30	−12.87	12.20	Demographic × Shape of Reproduction	0.16	0.01	0.14	0.18
Demographic × Parity [Semelparous]	0.22	6.19	−12.01	12.47					
Environmental × Parity [Iteroparous]	0.19	6.18	−11.89	11.57	Environmental × Shape of Reproduction	−0.22	0.01	−0.24	−0.19
Environmental × Parity [Semelparous]	0.10	6.14	−12.06	12.15					
Environ & Demo × Parity [Iteroparous]	−0.48	6.19	−12.59	11.38	Environ & Demo × Shape of Reproduction	0.05	0.01	0.03	0.08
Environ & Demo × Parity [Semelparous]	0.04	6.23	−12.45	12.16					
Asymptotic × Initial population size	0.04	0.02	0.01	0.07	Asymptotic × Initial population size	0.02	0.02	−0.01	0.05
Demographic × Initial population size	0.07	0.02	0.03	0.10	Demographic × Initial population size	0.09	0.02	0.05	0.12
Environmental × Initial population size	0.22	0.01	0.19	0.25	Environmental × Initial population size	0.19	0.01	0.16	0.22
Environ & Demo × Initial population size	−0.33	0.02	−0.37	−0.29	Environ & Demo × Initial population size	−0.30	0.02	−0.34	−0.26
Deviance	191258.80	6.16	191248.79	191272.58	Deviance	191978.68	6.23	191968.74	191993.10

### 3.2 Simulations of newly introduced populations

**FIGURE 3:**
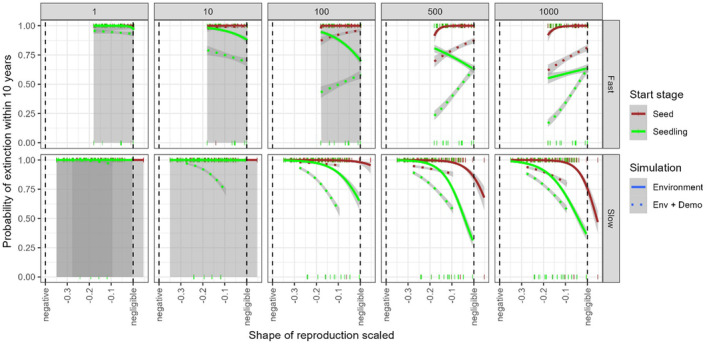
Variation in the probability of extinction within 10 years for finite populations with initial population sizes of 1, 10, 100, 500, or 1000 seed (red) or seedlings (green) as explained by the shape of reproduction (Baudisch & Stott, 2019). Shape≈0 indicates ‘negligible’ reproductive senescence; Shape<0 are semelparous with ‘negative’ reproductive senescence. Life histories are split by pace: fast (age of reproductive maturity <3 years) top row and slow (age of reproductive maturity ≥3 years) bottom row across five initial population sizes (columns). Line styles represent simulations of environmental stochasticity (solid), and both environmental and demographic stochastic processes (dotted) with shaded standard error. Line and tick colors represent populations founded with seed (dark red) or seedling (bright green). Tick marks show the distribution of extinctions (1) and persistence (0) within 10 simulated years for each founding stage. Extinction is defined as a population dropping below 1 individual.

The direction and magnitude of effects of some parameters on the odds of extinction for novel populations differed between short‐ (10 year) and long‐term (100 year) projection durations. Most notably, increasing the initial population size had a stronger effect over the long term of reducing the odds of extinction for populations initiated as seed but less of an impact over the short term while increasing the initial population size had a stronger effect of reducing the odds of extinction over the short‐term for populations initiated as seedlings but less of an effect over the long‐term (Appendix S1). The shape of reproduction (Equation 5a) was more strongly associated with variation in odds of extinction across founding populations size, founding population stage, pace, and stochasticity than was life history categories of reproductive lifespan (parity, Equation 5b; evidence ratio ≅∞). Pace did not influence the odds of extinction but with every standard deviation increase in shape (from negative to negligible senescence), there was a 27.9% (95% HPDI 26.8% to 29.0%) reduction in the odds of extinction (Figure 3). Stochasticity had a non‐zero effect on shape with an increase of one SD in shape resulting in a reduction in the odds of extinction of 20.5% for demographic stochasticity and 59.4% for demographic and environmental stochasticity but an increase in the odds of extinction of 11.3% for environmental stochasticity alone. Pace had a non‐zero influence on shape (Table 3b) with the reduction being more pronounced in fast (41%) while slow life histories had an increase of the odds of extinction (4.8%) with each SD increase towards negligible senescence.

**TABLE 3:** Log‐odds of extinction within 10 simulated years of novel populations initiated as either seed or seedlings (the first non‐dormant stage) for initial population sizes of [1, 10, 100, 500, or 1000] as explained by pace, founding population size, founding population stage (seed or seedlings), simulated stochasticity, and (a) parity (semelparous: <3 years of reproductive lifespan; iteroparity: ≥3 years of reproductive lifespan) or (b) shape of reproduction (Shape (Baudisch & Stott, 2019) negative senescence, ${\text{Shape}}_{\min}=-0.375$, to negligible senescence, Shape = 0, and towards positive senescence, ${\text{Shape}}_{\max}=0.14$), and their interactions. Parameter estimates, mean, lower and upper highest posterior density interval and standard deviation, are presented on the logit scale and represent the difference from the mean of each parameter. Starting population size and shape of reproduction were scaled and centered. Model fit using JAGS, 3 chains each with 10,000 iterations (first 5000 discarded), thinning to every sixth step. Non‐zero effects are highlighted.

(a) Categorical parity–duration	Mean	SD	Lower HPDI 2.50%	Upper HPDI 97.50%	(b) Continuous shape–intensity	Mean	SD	Lower HPDI 2.50%	Upper HPDI 97.50%
					DIC: 178,377.2				
(Intercept)	−0.06	0.01	−0.08	−0.05	(Intercept)	0.17	0.01	0.15	0.19
Pace [Fast]	−0.10	5.30	−10.28	10.34	Pace [Fast]	−0.27	4.74	−9.79	8.99
Pace [Slow]	0.10	5.30	−10.34	10.28	Pace [Slow]	0.27	4.74	−8.99	9.79
Parity [Iteroparous]	0.05	5.29	−10.25	9.99	Shape of Reproduction	−0.33	0.01	−0.34	−0.31
Parity [Semelparous]	−0.05	5.29	−9.99	10.25					
Initial population size	−1.36	0.01	−1.37	−1.35	Initial population size	−1.66	0.01	−1.68	−1.64
Founding stage [Seed]	0.29	5.23	−9.92	10.53	Founding stage [Seed]	0.74	4.67	−8.65	9.74
Founding stage [Seedling]	−0.29	5.23	−10.53	9.92	Founding stage [Seedling]	−0.74	4.67	−9.74	8.65
Asymptotic	−0.07	6.55	−13.14	12.39	Asymptotic	0.34	6.07	−11.35	12.00
Demographic Stochasticity	−0.26	6.86	−13.53	13.44	Demographic Stochasticity	−0.41	6.09	−12.77	11.28
Environmental Stochasticity	0.46	6.76	−12.72	13.07	Environmental Stochasticity	0.59	6.17	−11.11	12.69
Environmental and Demographic Stochasticity	−0.13	6.63	−13.22	12.63	Environmental and Demographic Stochasticity	−0.53	6.21	−12.68	11.81
Pace [Fast] × Parity [Iteroparous]	−0.29	6.27	−12.38	12.12	Pace [Fast] × Shape of Reproduction	−0.05	0.01	−0.06	−0.03
Pace [Slow] × Parity [Iteroparous]	0.76	6.21	−11.47	12.49					
Pace [Fast] × Parity [Semelparous]	−0.16	6.21	−11.89	12.06	Pace [Slow] × Shape of Reproduction	0.05	0.01	0.03	0.06
Pace [Slow] × Parity [Semelparous]	−0.31	6.27	−12.71	11.79					
Pace [Fast] × Initial population size	−0.04	0.01	−0.05	−0.03	Pace [Fast] × Initial population size	0.04	0.01	0.02	0.06
Pace [Slow] × Initial population size	0.04	0.01	0.03	0.05	Pace [Slow] × Initial population size	−0.04	0.01	−0.06	−0.02
Pace [Fast] × Founding stage [Seed]	0.23	6.18	−11.62	12.35	Pace [Fast] × Founding stage [Seed]	0.18	5.88	−11.32	11.98
Pace [Slow] × Founding stage [Seed]	0.21	6.25	−11.93	12.65	Pace [Slow] × Founding stage [Seed]	0.46	6.05	−11.18	11.97
Pace [Fast] × Founding stage [Seedling]	−0.34	6.25	−12.78	11.81	Pace [Fast] × Founding stage [Seedling]	−0.20	6.05	−11.70	11.45
Pace [Slow] × Founding stage [Seedling]	−0.11	6.18	−12.21	11.74	Pace [Slow] × Founding stage [Seedling]	−0.44	5.88	−12.23	11.08
Asymptotic × Pace [Fast]	0.11	6.42	−12.51	12.89	Asymptotic × Pace [Fast]	−0.07	6.16	−12.28	11.95
Asymptotic × Pace [Slow]	0.20	6.39	−12.83	12.41	Asymptotic × Pace [Slow]	0.04	6.22	−12.28	12.32
Demographic × Pace [Fast]	−0.25	6.35	−12.67	12.37	Demographic × Pace [Fast]	0.11	6.19	−12.10	12.28
Demographic × Pace [Slow]	−0.12	6.36	−12.53	12.23	Demographic × Pace [Slow]	0.05	6.30	−12.48	12.63
Environmental × Pace [Fast]	0.20	6.59	−13.18	12.80	Environmental × Pace [Fast]	0.27	6.32	−11.93	12.83
Environmental × Pace [Slow]	0.08	6.29	−12.25	12.22	Environmental × Pace [Slow]	0.15	6.16	−12.15	11.82
Environ & Demo × Pace [Fast]	−0.26	6.38	−12.77	12.16	Environ & Demo × Pace [Fast]	−0.37	6.18	−12.03	11.76
Environ & Demo × Pace [Slow]	0.04	6.70	−12.85	13.25	Environ & Demo × Pace [Slow]	−0.19	6.16	−11.99	12.27
Parity [Iteroparous] × Founding stage [Seed]	0.63	6.06	−11.11	12.75	Shape of Reproduction × Founding stage [Seed]	−0.21	0.01	−0.23	−0.20
Parity [Semelparous] × Founding stage [Seed]	−0.23	6.34	−12.29	12.76					
Parity [Iteroparous] × Founding stage [Seedling]	−0.27	6.34	−13.27	11.77	Shape of Reproduction × Founding stage [Seedling]	0.21	0.01	0.20	0.23
Parity [Semelparous] × Founding stage [Seedling]	−0.13	6.06	−12.27	11.61					
Parity [Iteroparous] × Initial population size	0.04	0.01	0.03	0.06	Initial population size × Shape of Reproduction	0.08	0.01	0.06	0.10
Parity [Semelparous] × Initial population size	−0.04	0.01	−0.06	−0.03					
Asymptotic × Parity [Iteroparous]	0.11	6.47	−12.03	12.56	Asymptotic × Shape of Reproduction	0.01	0.01	−0.01	0.03
Asymptotic × Parity [Semelparous]	−0.05	6.47	−12.61	12.98					
Demographic × Parity [Iteroparous]	−0.16	6.47	−12.80	12.20	Demographic × Shape of Reproduction	0.08	0.01	0.06	0.10
Demographic × Parity [Semelparous]	0.21	6.53	−12.35	12.63					
Environmental × Parity [Iteroparous]	0.26	6.37	−11.82	13.14	Environmental × Shape of Reproduction	0.08	0.01	0.05	0.11
Environmental × Parity [Semelparous]	−0.04	6.41	−12.70	12.70					
Environ & Demo × Parity [Iteroparous]	−0.10	6.38	−12.80	12.26	Environ & Demo × Shape of Reproduction	−0.17	0.02	−0.20	−0.14
Environ & Demo × Parity [Semelparous]	−0.23	6.28	−12.32	12.18					
Founding stage [Seed] × Initial population size	0.27	0.01	0.26	0.28	Founding stage [Seed] × Initial population size	0.57	0.01	0.55	0.59
Founding stage [Seedling] × Initial population size	−0.27	0.01	−0.28	−0.26	Founding stage [Seedling] × Initial population size	−0.57	0.01	−0.59	−0.55
Asymptotic × Initial population size	−0.06	0.01	−0.08	−0.04	Asymptotic × Initial population size	−0.04	0.01	−0.07	−0.02
Demographic × Initial population size	0.08	0.01	0.06	0.10	Demographic × Initial population size	0.11	0.01	0.09	0.14
Environmental × Initial population size	0.25	0.01	0.23	0.26	Environmental × Initial population size	0.15	0.01	0.12	0.18
Environ & Demo × Initial population size	−0.27	0.01	−0.29	−0.25	Environ & Demo × Initial population size	−0.22	0.02	−0.25	−0.19
Asymptotic × Founding stage [Seed]	0.13	6.25	−12.19	12.81	Asymptotic × Founding stage [Seed]	−0.08	6.22	−12.18	11.89
Asymptotic × Founding stage [Seedling]	−0.14	6.47	−12.93	12.15	Asymptotic × Founding stage [Seedling]	−0.13	6.14	−12.51	11.55
Demographic × Founding stage [Seed]	−0.09	6.49	−12.52	12.55	Demographic × Founding stage [Seed]	−0.32	6.16	−12.41	11.80
Demographic × Founding stage [Seedling]	−0.18	6.49	−13.06	12.29	Demographic × Founding stage [Seedling]	−0.22	6.18	−12.16	12.09
Environmental × Founding stage [Seed]	0.77	6.55	−12.21	14.00	Environmental × Founding stage [Seed]	0.66	6.16	−11.23	12.91
Environmental × Founding stage [Seedling]	−0.05	6.41	−12.46	12.42	Environmental × Founding stage [Seedling]	−0.02	6.24	−12.31	11.67
Environ & Demo × Founding stage [Seed]	0.01	6.56	−12.90	12.99	Environ & Demo × Founding stage [Seed]	0.29	6.26	−11.22	12.85
Environ & Demo × Founding stage [Seedling]	−0.46	6.48	−13.12	12.20	Environ & Demo × Founding stage [Seedling]	−0.18	6.23	−11.92	11.73
Deviance	377434.62	7.20	377422.88	377450.67	Deviance	178351.44	7.18	178338.91	178367.02

The addition of approximately 385 individuals to the founding population size reduced the odds of extinction by 80.9% (95% HPDI 80.6% to 81.3%). An increase of one SD in shape resulted in an 85% reduction in the odds of extinction for every SD increase in initial population size. The effect of an SD increase in initial population size results in a 68.4% drop in the odds of extinction for environmental stochasticity, a 75.1% drop for asymptotic conditions, 78.3% for demographic stochasticity, and an 89.8% drop for environmental and demographic stochasticity. The log‐odds of extinction do not vary by founding stage alone (Table 3b). The effects of the founding stage are not modified by pace or any stochastic treatment. Pace had a non‐zero effect on initial population size (Table 3b) with an increase to 79.9% reduction in the odds of extinction for an SD increase in initial population size for fast pace and a 74.5% reduction for slow pace when all other parameters were held at their mean. Under environmental and demographic stochasticity, the odds of extinction dropped by 90.9% for fast and 88.5% for slow life histories for every increase in an SD of initial population size. The effect of founding population size was mitigated by the founding stage whereas, for an increase in one SD of initial population size, the odds of extinction dropped by 94.3% for populations founded with seedlings but only by 10.4% for populations founded with seed (Figure 3). Under environmental and demogrpahic stochasticity, the odds of extinction dropped by 97.4% for populations founded with seedlings and 59.5% for populations founded with seed for each increase of one SD in initial population size.

## 4 DISCUSSION

Although aspects of short‐ compared to long‐term population dynamics independently influence odds of extinction of small populations (Iles et al., 2016), in our simulations, the direction and size of effects of life history, population size, and stochasticity on the odds of extinction did not differ between models of 10 and 100 years. Therefore, parameters important in long‐term dynamics are equally important to consider in the near term. This is fortunate because appropriate conservation actions must be implemented and tested over short time frames. Our results indicate that transient dynamics can be estimated using a species' life history. However, due to the rapid pace of biodiversity loss, many conservation actions must be implemented with incomplete species knowledge. Our results highlight which aspects of a species' life history best predict transient population dynamics given the population size, population history, and stochastic environment. Focusing data collection accordingly will improve the ability to apply appropriate conservation actions.

In both cases of population history, size matters most. For existing populations, population size has the greatest impact on reducing the odds of extinction under the realistic conditions of both demographic and environmental stochasticity. In existing populations, population size mattered more for slow than fast pace while among novel populations, size mattered more for fast than slow pace. The tradeoff between a quick evolutionary response to environmental change and demographic resilience to environmental stochasticity (Schmid et al., 2022) is a result of a species' pace and the fundamental tradeoff between survival and reproduction (Stearns, 1976). Fast species tend to be labile in vital rates resulting in big gains when conditions are favorable while slow species dampen population fluctuations by reducing vital rate responses to environmental variability (Koons et al., 2009; Le Coeur et al., 2022; Rodriguez‐Caro et al., 2021). Lability could explain why a larger population size does more to reduce the odds of extinction in novel populations of fast species. The potential of extinction is greater for fast species when all individuals are in vegetative or seed stages. Even in larger, existing populations, demographic stochasticity can lower population growth rates and slow evolutionary adaptive dynamics (Steiner et al., 2021). Small, existing populations are buffered from extirpation through the diversity of stage classes, but the effects of demographic stochasticity could explain why the effect of population size was greater for slow than fast species in existing populations. In our simulations, demographic stochasticity reduced the odds of extinction for semelparous life histories as the population size increased. Conversely, the odds of extinction for iteroparous life histories increased due to demographic stochasticity even as the population size increased. This pattern was likely due to lower variation in reproductive rates for iteroparous than semelparous strategies. However, these patterns are extrapolating behavior from the empirical data of potentially much larger populations. While these might not represent the population growth rates at low densities, these do provide a starting point to check deviations from expectations in future studies and conservation projects.

Two distinct reproductive traits, duration, and shape of reproduction, differed by population history in explaining variation in near‐term odds of extinction. Reproduction encompasses fecundity (number of offspring), parity, the shape of reproduction, and the allocation of resources towards seeds and seedlings (soil seed bank). Slow species avoid extinction through heavy investment in the survival of larger stages and low investment in offspring or dispersal (Bossuyt & Honnay, 2006). Slow species are thus less likely to form persistent soil seed banks than fast species (Honda, 2008; Rosbakh et al., 2022). Accordingly, we found high variability in soil seed bank persistence of both fast iteroparous and slow semelparous life history categories. The odds of extinction are spread over time through the survival of dormant seed in fast species and the survival of non‐dormant stages in slow species (Saatkamp et al., 2014). Environmental variability, specifically fluctuating temperatures, is required to break seed dormancy (Honda, 2008) which drives the rate of germination. In unpredictable environments, iteroparous individuals that consistently produce seed risk little from occasional reproductive failure while semelparous individuals that risk total reproductive failure instead spread risk over time through delayed germination of seed in the soil (Saatkamp et al., 2014).

Our results match previous findings that a high degree of iteroparity S≅0 is correlated to long‐term population growth while concentrated reproductive effort is associated with declining population growth rates (Salguero‐Gómez, 2017). A species with uniform reproductive intensity has the ability to spread risk of reproductive failure over time similarly to a species with a long reproductive lifespan. In our study, most population matrices with short reproductive lifespan also had a shape of reproduction S≅0. Iteroparous life histories spread reproductive efforts across many years but, in our analyses, generally continued to increase reproductive effort with age. In our simulations, the odds of extinction increased as the shape of reproduction approached −0.5 despite the duration of reproduction. This may be due to low fecundity rates early in an iteroparous lifespan which do less to spread risk over time and buffer against stochasticity. When reproductive efforts are concentrated, the risk is spread across individuals. For slow life histories, the risk is spread across (st)ages putting populations in unstable structures at greater risk.

The effect of population size on the odds of extinction is mitigated by life history traits. Therefore, life history can be used to set target population sizes to prevent extinction and invasions and improve conservation outcomes for reintroduction and restoration. Our study shows that by estimating a species' shape of reproduction, we can predict transient population dynamics to reduce the risk of plant invasions and increase the success of restoration. Our results also have implications in balancing the founding stage and number of individuals to increase restoration and reintroductions success. With an estimate of reproductive lifespan, we can apply appropriate population size targets for the recovery of small populations. In novel populations, our results show that increasing the number of seedlings reduces the odds of extinction more than increasing the number of seeds sown. Unfortunately, there are limited seeds for restoration and reintroductions, limited capacity to use a larger stage class (by germinating seeds ex‐situ and out planting a vegetative stage), and uncertainty around best restoration and reintroduction practices despite extensive research on how to maximize success (Albrecht & Maschinski, 2012; Shaw et al., 2020). To address these issues, a third important implication of our findings is that life history can be used to determining when efforts should be directed toward harvesting additional seeds or towards growing larger founding stages to improve the success of restoration and reintroduction projects. The practice of adding trees and shrubs (typically slow iteroparous species) as seedlings to add diversity with few individuals is supported by our simulations but more individuals are needed for an iteroparous than semelparous species to persist. By contrast, in our simulations, there was only a small gain in risk reduction by using seedlings for semelparous life histories. Semelparous species put the most resources towards producing many small seeds that can persist in the soil seed bank so reintroductions by seed may give these life histories the best chance of long‐term population persistence. Our simulations suggest that there are larger gains in using a non‐dormant founding stage when a greater number of individuals can be planted. When this is impractical, risk can be reduced through multiple plantings to increase variation in stage distribution of the population. Our results suggest that collecting seed from an iteroparous species will have little impact on population survival which might be due to lower investment in the seed stage compared to vegetative stages. Conversely, semelparous species may be able to withstand a higher level of seed collection than iteroparous species because they are robust to small population sizes and produce many seeds that persist in the soil seed bank.

Conservation planning hinges on the fundamental tradeoff between survival and reproduction using proxies of pace to predict demographic performance (Albrecht et al., 2019; Albrecht & Maschinski, 2012; Shaw et al., 2020). Here we have shown that the probability a population will persist or perish depends on how risk of reproductive failure is distributed over time or across individuals in stochastic environments with random variation among indiviudals. For species lacking demographic information, parity is a suitable shorthand for predicting population dynamics for populations with stable stage structure. However, when populations cannot spread risk across multiple stage classes as is the case in novel populations, the shape of reproduction is needed to predict the odds of extinction. We provide evidence that life history categories can be used to predict near‐term demographic performance of finite populations and be applied to combating plant invasions and improving rare plant conservation.

## AUTHOR CONTRIBUTIONS

**Michelle DePrenger‐Levin:** Conceptualization (lead); data curation (lead); formal analysis (lead); methodology (equal); writing – original draft (lead); writing – review and editing (equal). **Michael B. Wunder:** Conceptualization (equal); writing – review and editing (equal).

### OPEN RESEARCH BADGES

This article has earned Open Data and Open Materials badges. Data and materials are available at https://github.com/DenverBotanicGardens/TraitsExtinctionRisk.

## Supporting information


Appendix S1.

## Data Availability

If the manuscript is accepted, the subset of matrix population models collected from the COMPADRE plant matrix database and additional metrics derived from the matrices are deposited in DRYAD (https://doi.org/10.5061/dryad.2547d7wzv). Code (in R and JAGS) used to run the analyses and create the figures are accessible in a public Git repository https://github.com/DenverBotanicGardens/TraitsExtinctionRisk.

## References

[ece311044-bib-0001] Albrecht, M. , & Maschinski, J. (2012). Influence of founder population size, propagule stages, and life history on the survival of reintroduced plant populations. In J. Maschinski , K. Haskins , & P. Raven (Eds.), Plant reintroduction in a changing climate: Promises and perils (pp. 171–188). Island Press.

[ece311044-bib-0002] Albrecht, M. A. , Osazuwa‐Peters, O. L. , Maschinski, J. , Bell, T. J. , Bowles, M. L. , Brumback, W. E. , Duquesnel, J. , Kunz, M. , Lange, J. , McCue, K. A. , McEachern, A. K. , Murray, S. , Olwell, P. , Pavlovic, N. B. , Peterson, C. L. , Possley, J. , Randall, J. L. , & Wright, S. J. (2019). Effects of life history and reproduction on recruitment time lags in reintroductions of rare plants. Conservation Biology, 33, 601–611. 30461065 10.1111/cobi.13255

[ece311044-bib-0003] Baudisch, A. , & Stott, I. (2019). A pace and shape perspective on fertility. Methods in Ecology and Evolution, 10, 1941–1951.

[ece311044-bib-0004] Bossuyt, B. , & Honnay, O. (2006). Interactions between plant life span, seed dispersal capacity and fecundity determine metapopulation viability in a dynamic landscape. Landscape Ecology, 21, 1195–1205.

[ece311044-bib-0005] Caswell, H. (2001). Matrix population models: Construction, analysis, and interpretation. Sinauer Associates.

[ece311044-bib-0006] Caswell, H. , & Salguero‐Gomez, R. (2013). Age, stage and senescence in plants. Journal of Ecology, 101, 585–595. 23741075 10.1111/1365-2745.12088 PMC3664411

[ece311044-bib-0007] Chamberlain, S. A. , Hovick, S. M. , Dibble, C. J. , Rasmussen, N. L. , van Allen, B. G. , Maitner, B. S. , Ahern, J. R. , Bell‐Dereske, L. P. , Roy, C. L. , Meza‐Lopez, M. , Carrillo, J. , Siemann, E. , Lajeunesse, M. J. , & Whitney, K. D. (2012). Does phylogeny matter? Assessing the impact of phylogenetic information in ecological meta‐analysis. Ecology Letters, 15, 627–636. 22487445 10.1111/j.1461-0248.2012.01776.x

[ece311044-bib-0008] Cochran, M. E. , & Ellner, S. (1992). Simple methods for calculating age‐based life‐history parameters for stage‐structured populations. Ecological Monographs, 62, 345–364.

[ece311044-bib-0009] Crawley, M. J. (1996). Life history and environment. In Plant ecology (pp. 73–131). John Wiley & Sons, Ltd.

[ece311044-bib-0010] Demetrius, L. (1978). Adaptive value, entropy and survivorship curves. Nature, 275, 213–214. 692692 10.1038/275213a0

[ece311044-bib-0011] Ellis, M. M. , & Crone, E. E. (2013). The role of transient dynamics in stochastic population growth for nine perennial plants. Ecology, 94, 1681–1686. 24015512 10.1890/13-0028.1

[ece311044-bib-0012] Guo, Y. , Lu, W. , Che, Z. , Cao, J. , Yang, H. , & Huang, X. (2022). A meta‐analysis on the relationship between seed size, seed shape and persistence in soil seed bank. Pakistan Journal of Botany, 54, 925–930.

[ece311044-bib-0013] Honda, Y. (2008). Ecological correlations between the persistence of the soil seed bank and several plant traits, including seed dormancy. Plant Ecology, 196, 301–309.

[ece311044-bib-0014] Iles, D. T. , Salguero‐Gómez, R. , Adler, P. B. , & Koons, D. N. (2016). Linking transient dynamics and life history to biological invasion success. Journal of Ecology, 104, 399–408.

[ece311044-bib-0015] IPCC . (2022). Climate change 2022: Impacts, adaptation and vulnerability. In H.‐O. Pörtner , D. C. Roberts , M. Tignor , E. S. Poloczanska , K. Mintenbeck , A. Alegría , M. Craig , S. Langsdorf , S. Löschke , V. Möller , A. Okem , & B. Rama (Eds.), Contribution of working group II to the sixth assessment report of the intergovernmental panel on climate change (pp.3056). Cambridge University Press. 10.1017/9781009325844

[ece311044-bib-0016] Jeppsson, T. , & Forslund, P. (2012). Can life history predict the effect of demographic Stochasticity on extinction risk? American Naturalist, 179, 706–720. 10.1086/665696 22617260

[ece311044-bib-0017] Jones, O. R. , Barks, P. , Stott, I. , James, T. D. , Levin, S. , Petry, W. K. , Capdevila, P. , Che‐Castaldo, J. , Jackson, J. , Römer, G. , Schuette, C. , Thomas, C. C. , & Salguero‐Gómez, R. (2022). Rcompadre and rage‐two R packages to facilitate the use of the COMPADRE and COMADRE databases and calculation of life‐history traits from matrix population models. Methods in Ecology and Evolution, 13, 770–781.

[ece311044-bib-0018] Kendall, B. E. (1998). Estimating the magnitude of environmental stochasticity in survivorship data. Ecological Applications, 8, 184–193.

[ece311044-bib-0019] Kokko, H. , & Ebenhard, T. (1996). Measuring the strength of demographic stochasticity. Journal of Theoretical Biology, 183, 169–178.

[ece311044-bib-0020] Koons, D. N. , Iles, D. T. , Schaub, M. , & Caswell, H. (2016). A life‐history perspective on the demographic drivers of structured population dynamics in changing environments. Ecology Letters, 19, 1023–1031. 27401966 10.1111/ele.12628

[ece311044-bib-0021] Koons, D. N. , Pavard, S. , Baudisch, A. , & Metcalf, C. J. E. (2009). Is life‐history buffering or lability adaptive in stochastic environments? Oikos, 118, 972–980.

[ece311044-bib-0022] Le Coeur, C. , Yoccoz, N. G. , Salguero‐Gomez, R. , & Vindenes, Y. (2022). Life history adaptations to fluctuating environments: Combined effects of demographic buffering and lability. Ecology Letters, 25, 2107–2119. 35986627 10.1111/ele.14071 PMC9804727

[ece311044-bib-0023] Parry, G. D. (1981). The meanings of r‐selection and K‐selection. Oecologia, 48, 260–264. 28309810 10.1007/BF00347974

[ece311044-bib-0024] Perkins, D. L. , Parks, C. G. , Dwire, K. A. , Endress, B. A. , & Johnson, K. L. (2006). Age structure and age‐related performance of sulfur cinquefoil (*Potentilla recta*). Weed Science, 54, 87–93.

[ece311044-bib-0025] Picó, F. X. , & Retana, J. (2008). Age‐specific, density‐dependent and environment‐based mortality of a short‐lived perennial herb. Plant Biology, 10, 374–381. 18426484 10.1111/j.1438-8677.2008.00044.x

[ece311044-bib-0026] Plummer, M. (2003). JAGS: A program for analysis of Bayesian graphical models using Gibbs sampling. Proceedings of the 3rd international workshop on distributed statistical computing (DSC 2003); Vienna, Austria.

[ece311044-bib-0027] Purchase, C. F. , Rooke, A. C. , Gaudry, M. J. , Treberg, J. R. , Mittell, E. A. , Morrissey, M. B. , & Rennie, M. D. (2022). A Synthesis of senescence predictions for indeterminate growth, and support from multiple tests in wild lake trout. Proceedings of the Royal Society B: Biological Sciences, 289, 289. 10.1098/rspb.2021.2146 PMC8727146 34982951

[ece311044-bib-0028] R Core Team . (2022). R: A language and environment for statistical computing. R Foundation for Statistical Computing.

[ece311044-bib-0029] Rodriguez‐Caro, R. C. , Capdevila, P. , Gracia, E. , Barbosa, J. M. , Gimenez, A. , & Salguero‐Gomez, R. (2021). The limits of demographic buffering in coping with environmental variation. Oikos, 30, 1346–1358.

[ece311044-bib-0030] Rosbakh, S. , Pichler, M. , & Poschlod, P. (2022). Machine‐learning algorithms predict soil seed bank persistence from easily available traits. Applied Vegetation Science, 25, e12660.

[ece311044-bib-0031] Saatkamp, A. , Poschlod, P. , & Venable, D. L. (2014). The functional role of soil seed banks in natural communities. In R. S. Gallagher (Ed.), Seeds: The ecology of regeneration in plant communities. 3rd ed., 263–295. CABI.

[ece311044-bib-0032] Salguero‐Gómez, R. (2017). Applications of the fast–slow continuum and reproductive strategy framework of plant life histories. New Phytologist, 213, 1618–1624. 27864957 10.1111/nph.14289

[ece311044-bib-0033] Salguero‐Gomez, R. , Jones, O. R. , Archer, C. R. , Buckley, Y. M. , Che‐Castaldo, J. , Caswell, H. , et al. (2015). The COMPADRE plant matrix database: An open online repository for plant demography. Journal of Ecology, 103, 202–218.

[ece311044-bib-0034] Salguero‐Gomez, R. , Jones, O. R. , Jongejans, E. , Blomberg, S. P. , Hodgson, D. J. , Mbeau‐Ache, C. , et al. (2016). Fast‐slow continuum and reproductive strategies structure plant life‐history variation worldwide. Proceedings of the National Academy of Sciences of the United States of America, 113, 230–235. 26699477 10.1073/pnas.1506215112 PMC4711876

[ece311044-bib-0035] Salguero‐Gomez, R. , & Plotkin, J. B. (2010). Matrix dimensions bias demographic inferences: Implications for comparative plant demography. American Naturalist, 176, 710–722. 10.1086/657044 20964622

[ece311044-bib-0036] Schmid, M. , Paniw, M. , Postuma, M. , Ozgul, A. , & Guillaume, F. (2022). A trade‐off between robustness to environmental fluctuations and speed of evolution. American Naturalist, 200, 16–35. 10.1086/719654 35737989

[ece311044-bib-0037] Shaw, N. , Barak, R. S. , Campbell, R. E. , Kirmer, A. , Pedrini, S. , Dixon, K. , & Frischie, S. (2020). Seed use in the field: Delivering seeds for restoration success. Restoration Ecology, 28, S276–S285.

[ece311044-bib-0038] Simberloff, D. (2009). The role of propagule pressure in biological invasions. Annual Review of Ecology, Evolution, and Systematics, 40, 81–102.

[ece311044-bib-0039] Stearns, S. (1976). Life history tactics: A review of the ideas. The Quarterly Review of Biology, 51, 3–47. 778893 10.1086/409052

[ece311044-bib-0040] Steiner, U. K. , Tuljapurkar, S. , & Roach, D. A. (2021). Quantifying the effect of genetic, environmental and individual demographic stochastic variability for population dynamics in Plantago lanceolata. Scientific Reports, 11, 23174. 34848768 10.1038/s41598-021-02468-9 PMC8633285

[ece311044-bib-0041] Stott, I. , Townley, S. , & Hodgson, D. J. (2011). A framework for studying transient dynamics of population projection matrix models. Ecology Letters, 14, 959–970. 21790932 10.1111/j.1461-0248.2011.01659.x

[ece311044-bib-0042] Terry, J. C. D. , O'Sullivan, J. D. , & Rossberg, A. G. (2022). Synthesising the multiple impacts of climatic variability on community responses to climate change. Ecography, 2022, e06123.

[ece311044-bib-0043] Thurman, L. L. , Stein, B. A. , Beever, E. A. , Foden, W. , Geange, S. R. , Green, N. , Gross, J. E. , Lawrence, D. J. , LeDee, O. , Olden, J. D. , Thompson, L. M. , & Young, B. E. (2020). Persist in place or shift in space? Evaluating the adaptive capacity of species to climate change. Frontiers in Ecology and the Environment, 18, 520–528.

