# Supplementary material for: Pace and parity predict the short‐term persistence of small plant populations
Source: Ecol Evol. 2024 Feb 20;14(2):e11044. doi: 10.1002/ece3.11044 (PMC10877661; doi:10.1002/ece3.11044)
Supplement: Supplementary file 1 — Appendix S1. [file ECE3-14-e11044-s001.docx]

Appendix S1

Michelle DePrenger-Levin

Phylogenetic relatedness

The phylogenetic relatedness of species is a potential source of nonindependence among life history traits (Chamberlain et al., 2012) and could explain variation in the odds of extinction within and among life-history categories and impact the magnitude or direction of effect sizes of pace, parity, population size, or stochastic process. Observed patterns of extinction risk by life-history traits could be due to distantly related lineages having similar evolutionary responses to similar enviornmental conditions or similar phenotypic solutions arising from common descent (Vasconcelos, 2023). As an example of the former, most slow and all iteroparous life histories are found near the equator for the full set of matrices used in the analysis (Figure S1) and the subset that have a seed stage (Figure S2). The latter is evident among the 17 of 46 families where all matrices of multiple species fall within one life history category. However, for 2/3 of the families with multiple species, populations with common descent have evolved different life history traits (Figure S3). Of the 73 families, 37 are represented by a single species, 18 by only 2 species, and 18 are represented by 3 or more species (Table S1). Among the most species rich families in our analysis, only Arecaceae, the palm family, are all slow iteroparous life-histories.

Many of the matrices, which represent the demographic performance under conditions of a particular year or location, may not accurately reflect the life history of that species as a whole. We observed that individual population matrix models of a species might respond to local enviornmental conditions in a given year or location by delaying or speeding up the age of reproductive maturity and decreasing or increasing adult longevity thereby altering pace or parity life history traits. Some matrices produced extreme estimates of age of maturity or adult longevity because a single matrix reflect the conditions of a single year in a single location and not the average behavior (the mean of all matrices) of a species. Therefore, while a single matrix does not always reflect the average demographic performance of a species, they do reflect observed correlations among vital rates that represent possible pace and parity strategies. Therefore, there may not be a need to correct for nonindependent evolutionary origins because these responses to the current environment indicate an absence of phylogenetic inertia (Felsenstein, 1985).

However, we attempted to understand the potential for nonindependence among our sampled taxa by creating a phylogeny. To account for phylogenetic relatedness among species within each life history category, we generated a plant phylogeny of 314 species available in Phylomatic version 3 (<http://phylodiversity.net/phylomatic/>) out of the 317 species used the simulations (Figure S4). We selected Scenario 3 which uses the PhytoPhylo megaphylogeny as a backbone and the branch length adjustment function implemented in Phylocom to estimate phylogenetic relatedness (Qian & Jin, 2016). Three species from the COMPADRE plants database were not available in the megaphylogeny, *Cystoseira zosteroides* an alga in the Sargassaceae family, *Laminaria digitata*, a kelp in the Laminariaceae family, and *Vulpicida pinastri*, a lichen in the Parmeliaceae family. We explored patterns of phylogenetic relatedness, clustering, and overdispersion among and within life history category using the R package *Picante* (Kembel et al., 2010). There is evidence of phylogenetic clustering within the slow iteroparous group (net relatedness index = -2.86) compared to phylogenetic overdispersion within the fast semelparous (NRI = 2.16), fast iteroparous (NRI = 1.72), and slow semelparous (NRI = 2.65). However, there are far more slow iteroparous species than species within the other categories. Because the phylogeny did not cover all species used the in analysis and is a rough estimate of phylogenetic relatedness, we instead explored how the magnitude of the effects varied with taxonomic identity using plant family as a grouping factor. Mode of reproduction and pace are well conserved among some families while others contain a mixture of life history traits (Figure S3).

We compared models of the impacts of life history, demographic and environmental stochasticity, population size, and in the case of novel populations, founding stage on near-term extinction risk with and without the group level effect of plant family (allowing a distinct intercept for each plant family) and found little difference in results when using this taxonomic rank to account for nonindependence among life history traits due to phylogenetic relatedness.

## Life history categorizations

A common proxy of pace is generation time because it measures the average age of reproductive adults at birth, the pace of evolution, and the time for a stable population to grow by the net reproductive rate (Caswell, 2001; Cochran & Ellner, 1992). Along with fecundity, generation time is predictive of extinction risk (Chichorro et al., 2019) and often used to normalize risk comparisons among diverse species. A shorter generation time means a greater evolutionary response to environmental change but greater sensitivity to short-term environmental variability (Schmid et al., 2022). The generation time of an iteroparous species that evenly allocates energy towards reproduction after reaching the age of reproductive maturity will be longer than the age of reproductive maturity while a semelparous species that waits until growth is maximized before switching to reproductive allocation will have a nearly identical generation time as the age of maturity (Baudisch & Stott, 2019; Figure S5a). Thus, to explore the effects on extinction risk of pace independent of parity, we used the age of reproductive maturity. The age of reproductive maturity better describes transient dynamics while generation time depends on the mode of reproduction and better describes long-term, asymptotic dyanmics.

The variation in vital rates within and among life history categories were examined to determine how well these generalizations capture a species’ response to demographic and environmental stochasticity. A species’ life history describes the timing, intensity, and duration of survival and reproduction. Life history determines how risk of extinction is spread over time, to cope with environmental stochasticity, or among individuals, in response to demographic stochasticity. Population stability and higher growth rates are predicted for slow life histories through demographic buffering (Koons et al., 2016) and iteroparous mode of reproduction through higher net reproductive rates (Salguero-Gómez, 2017). Our categorizations of matrix population models by life history strategy behaved as expected with the most variable growth rates among fast semelparous and the least variable among slow iteroparous life histories (Figure S5b).

To examine the dynamics of novel populations initiated as either seed or seedling, we used the R package *Rage* (Jones et al., 2022) to collapsed matrices with a seed stage into three stages: seed, vegetative, and reproductive. We quantified differences among life history categories in the allocation of resources for the vital rates of growth, stasis, maturation, seed survival, and seed germination (Figure S5c). The thresholds selected to define pace and parity resulted in expected allocations towards vital rates (Figure S5c). All life history categories had low germination rates with fast iteroparous life histories at the bottom. This confirms observations that iteroparous species produce few but larger seed in each reproductive effort and larger seed are less likely to form a persistent soil seed bank (Guo et al., 2022). Slow iteroparous life histories had the lowest seed survival rates among the four strategies and the highest reproductive stage survival, which agrees with findings that slow strategies spread risk over time through higher rates of juvenile and adult survival while fast species spread risk over time through a persistent soil seed bank (Saatkamp et al., 2014). Fast semelparous life histories had the highest rate of soil seed bank persistence confirming findings that competition among sibling seedings and bet hedging in variable environments is a strong evolutionary driver of delayed germination and variable germination percentages (Saatkamp et al., 2014). Iteroparous life histories had higher rates of reproductive survival than semelparous life histories and ‘semelparous’ reproductive survival was non-zero because semelparity was defined as adults surviving fewer than three years. Slow semelparous life histories had the highest rates of retrogression (transitioning from reproductive to non-reproductive) but this could be caused by clonal growth treated as survival instead of asexual reproduction (Janovsky et al., 2017). Fast semelparous life histories conversely put little energy towards survival of vegetative individuals and greater energy towards seeds that persist in the soil seed bank (Figure 1c).

The categorized mode of reproduction defines the duration of reproductive stage survival with iteroparity having the ability to spread risk of reproductive failure over time while semelparity concentrates reproduction over a short time. The continuous measure of reproductive effort defines how the intensity of reproductive effort is spread across the duration of reproductive survival. Consistent reproductive effort regardless of age ($S\cong0$) maximizes bet hedging against reproductive failure while reproductive output that increases with age concentrates reproductive effort over a shorter time frame. Among semelparous life histories (short duration), $S$ is concentrated around zero (mean +/- SD for fast $S=$ -0.052 +/- 0.072 and slow $S=$ -0.099 +/- 0.066) which indicates constant reproductive effort (Figure 2a). Life histories categorized as iteroparous (long duration) have shape values indicating increasing reproductive effort with age (fast mean +/- SD $S=$ -0.118 $+/-$ 0.058; slow mean +/- SD $S=$ -0.182 +/- 0.092; Figure 2b). While categorized life histories describe expected demographic performance, the categorical duration of reproductive effort alone does not fully describe the reproductive risk mitigating strategies of a life history.

In our analyses, we categorized parity by the duration of reproduction ($\alpha_{adultlong}$ adult longevity) and called those with a short duration ‘semelparous’ and with a long duration ‘iteroparous’. However, ‘semelparous’ means reproduction is followed by death. We compared models of extinction risk by pace and parity founded with seed or seedlings for parity categorized by reproductive duration longer or shorter than three years to one year. The threshold of three years was better at explaining variation in extinction risk (evidence ratio of 7.45) than one year, so we kept the threshold at three years of adult longevity.

The 10,000 matrices sampled for simulating population dynamics under asymptotic conditions had a median age of reproductive maturity of 4.64 years with a range of 1 – 10,010 years and a mean shape of reproduction of -0.135 (SD 0.097) with a range of -0.375 to 0.14.

Transient vs. long-term extinction risk

In both exisiting (Tables S2 and S3) and novel (Tables S4 and S5) population histories, simulations running 100 years better explain variation in the odds of extinction than 10 years. However, the typical research study or conservation plan may extend out 10 years and are unable to plan for 100 years. The impacts of initial population size, pace, duration and shape of reproduction, and stochasticity were similar between short- (10 years) and long-term dynamics (100 years), but important predictors differed.

For existing populations, many extinctions do not occur within 10 years, so each standard deviation unit increase in initial population size (ca. 385 individuals), results in a much larger reduction in the odds of extinction within 10 compared to 100 years. However, the only effect that changed direction between 10 and 100 years was that of environmental stochasticity on the effect of a SD increase in initial population size. With all other variables held at their mean and under asymptotic conditions, the effect of a SD increase in initial population size was an 83.4% reduction in the odds of extinction. Within 10 years and under environmental stochasticity alone, this effect was to reduce to an 80.1% reduction in the odds of extinction. Within 100 years, the interaction of initial population and environmental stochasticity was to increase the effect of a SD increase in initial population size from 47.4% reduction under asymptotic conditions to a 56.4% reduction in the odds of extinction under environmental stochasticity. Environmental stochasticity must lengthen the time to extinction resulting in fewer extinctions within 10 years than would be expected within 100 years.

For novel populations initiated as either only seed or only seedlings, fast life histories initiated as a single seed and simulated under asymptotic conditions are 30% more likely to go extinct within 100 years than 10 years (73% within 10 years, 96% within 100 years). Those initiated as seedlings are 167% more likely to go extinct within 100 than 10 years (29% within 10 years, 78% within 100 years). Restoration with seedlings may appear less likely to fail during a monitoring period but could still go extinct in the future. Slow life histories initiated as a single seed simulated under asymptotic conditions are only 12% more likely to go extinct within 100 than 10 years because extinction is likely for a single seed of a slow life history (87% within 10 years, 98% within 100 years). The probability that a single seedling of a slow life history drops such that the probability of extinction is 73% more like to go extinct within 100 than 10 years (51% within 10 years, 88% within 100 years). For each unit increase in shape of reproduction (towards iteroparity/negligible senescence) there is only a 0.6% increase in the probability of extinction within 10 years for slow life histories started as seed but a 47% increase in the probability of extinction for each unit increase in shape of reproduction within 10 years for slow life histories started as seedlings. Within 100 years one unit increase in shape of reproduction has nearly no impact on the probaility of extinction for a slow life history started as seed (0.01% increase for each unit increase in shape of reproduction) compared to 11% increase in risk of extinction for each unit increase in shape of reproduction for a slow life history started as a seedling.

Figure S1: Locations of matrix population models used in analyses. Color by life history strategy where fast have an age of reproductive maturity at fewer than three years and slow have an age of reproductive maturity of three or more years. Semelparous have adult longevity of fewer than three years and iteroparous have adult longevity of three or more years.


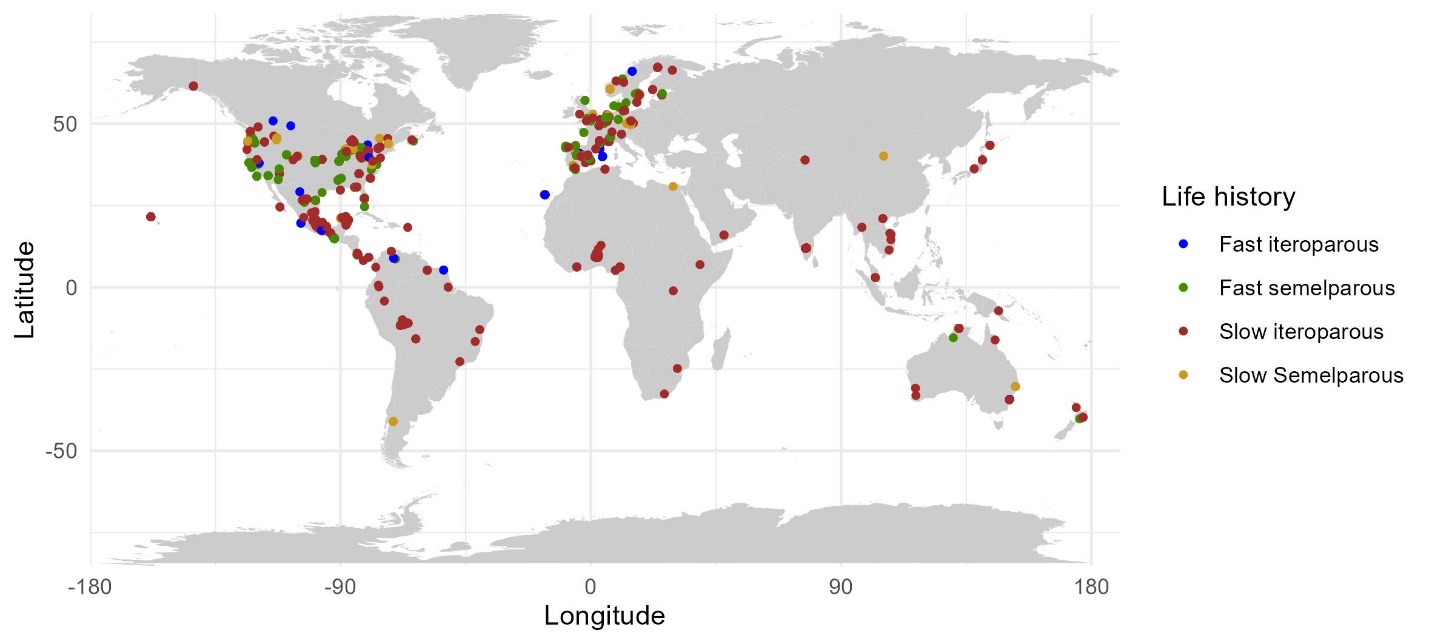


Figure S2: Locations of matrix population models with a seed stage. Color by life history strategy where fast have an age of reproductive maturity at fewer than three years and slow have an age of reproductive maturity of three or more years. Semelparous have adult longevity of fewer than three years and iteroparous have adult longevity of three or more years.


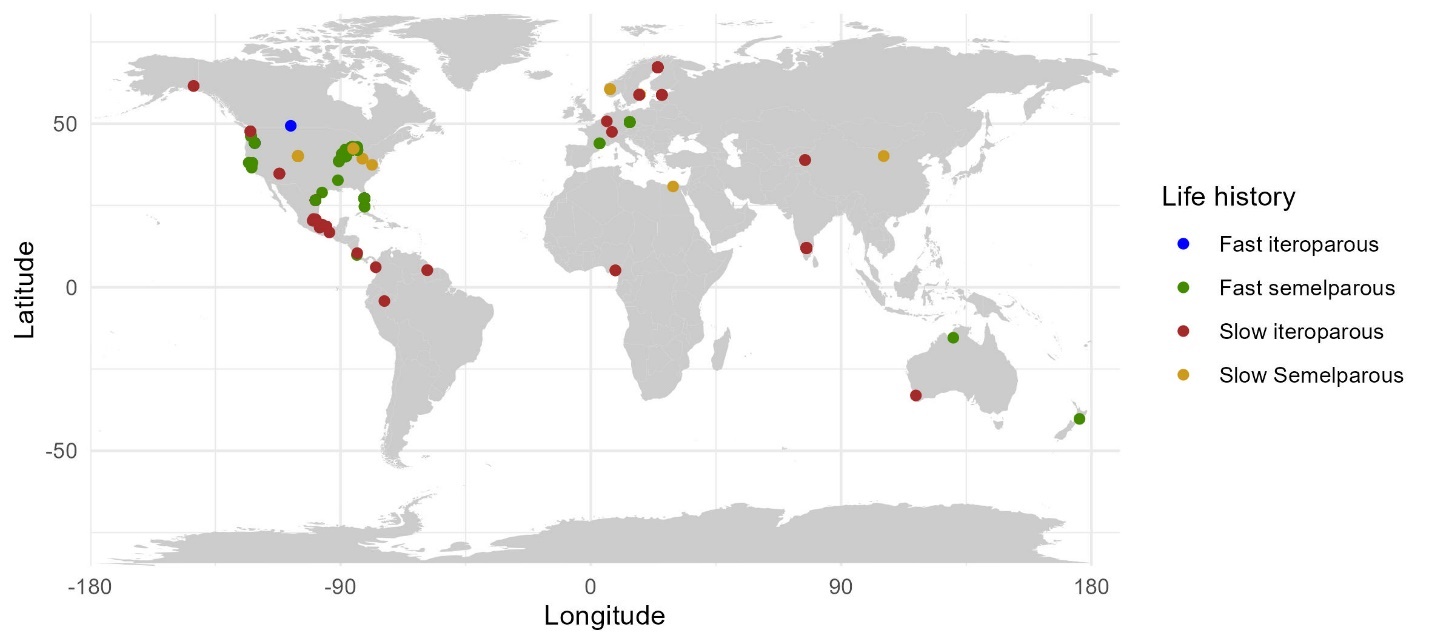


Figure S3: Number of species within each plant family in the analysis. Color by life history strategy where fast have an age of reproductive maturity at fewer than three years and slow have an age of reproductive maturity of three or more years. Semelparous have adult longevity of fewer than three years and iteroparous have adult longevity of three or more years. FI: fast iteroparous, FS: fast semelparous, SI: slow iteroparous, SS: slow semelparous.


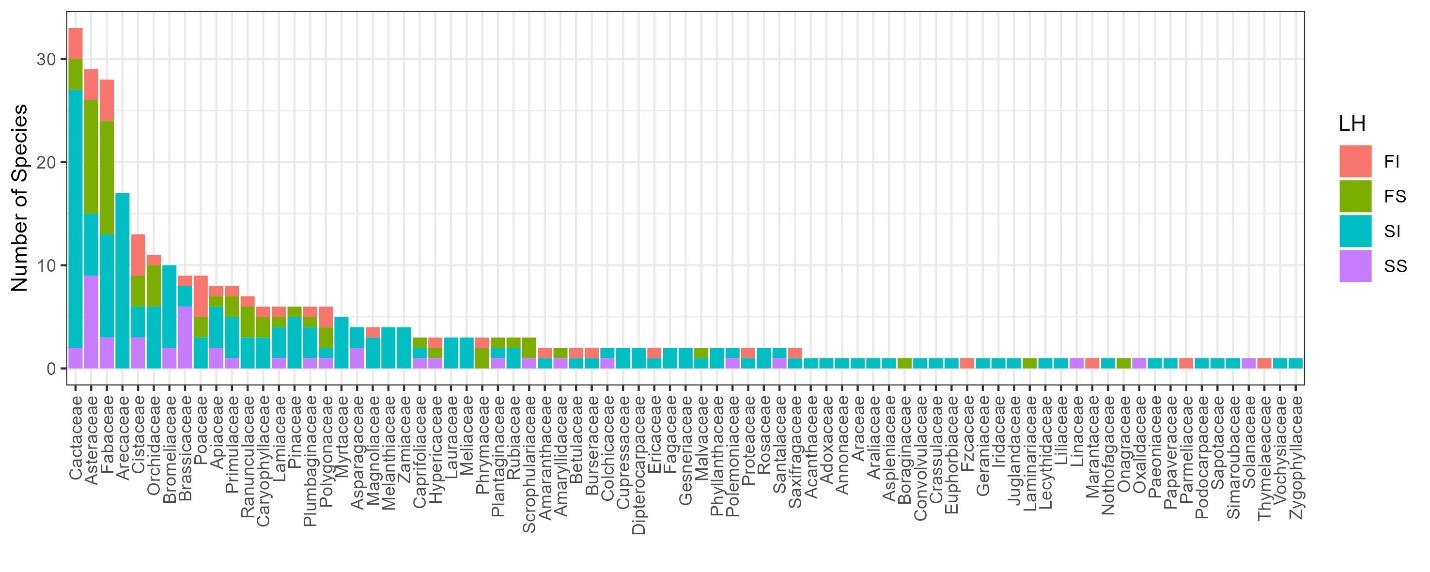


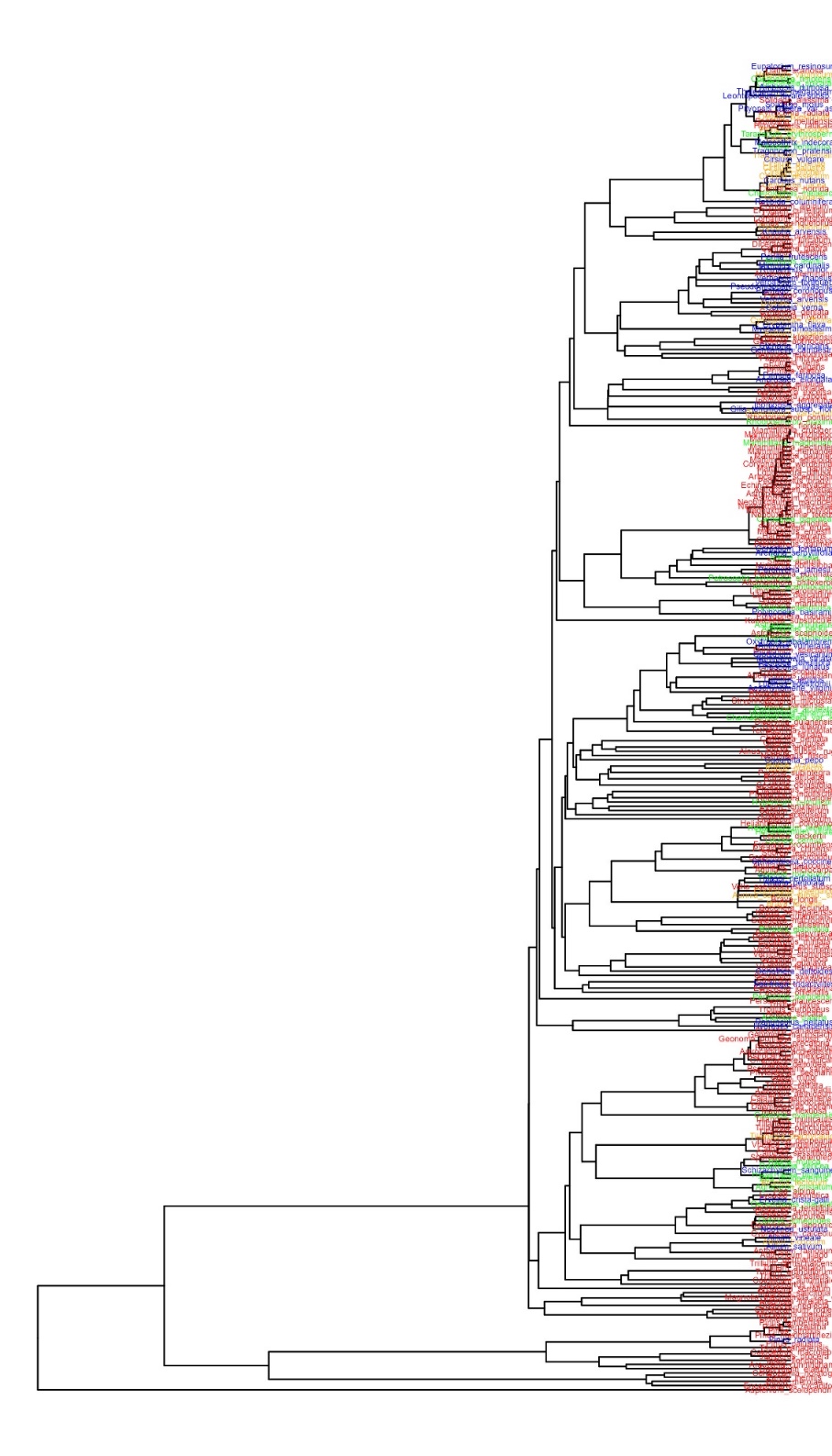


Figure S4. A phylogenetic tree based on the Phylomatic version 3 (<http://phylodiversity.net/phylomatic/>) PhytoPhylo megaphyogeny backbone of 314 species. Color represents life history category, red = slow iteroparous, blue = fast semelparous, green = fast iteroparous, and orange = slow semelparous.

Figure S5: a) Generation time of 1,606 stage-based population matrices representing 317 species from the COMPADRE plants matrix database (COMPADRE, 2022) in relation to their mean age of reproductive maturity. ‘Fast’ pace populations have an age of reproductive maturity $< 3$ years; ‘slow’ pace have an age of reproductive maturity of $\geq3$. Semelparous duration of reproduction have adult longevity $< 3$ years while iteroparous spend $\geq3$ years as reproductive adults. b) Log transformed population growth rates for each life history category; $\log\left( \lambda\right)=0$ is a stable, $>0$ is a growing, and $<0$ is a decreasing population. c) Life cycles of populations with a seed stage (472 matrices from 56 species in 28 families) condensed to a single seed, vegetative, and reproductive stage. Probability density function of the distribution of survival and growth transition rates for each life history category.


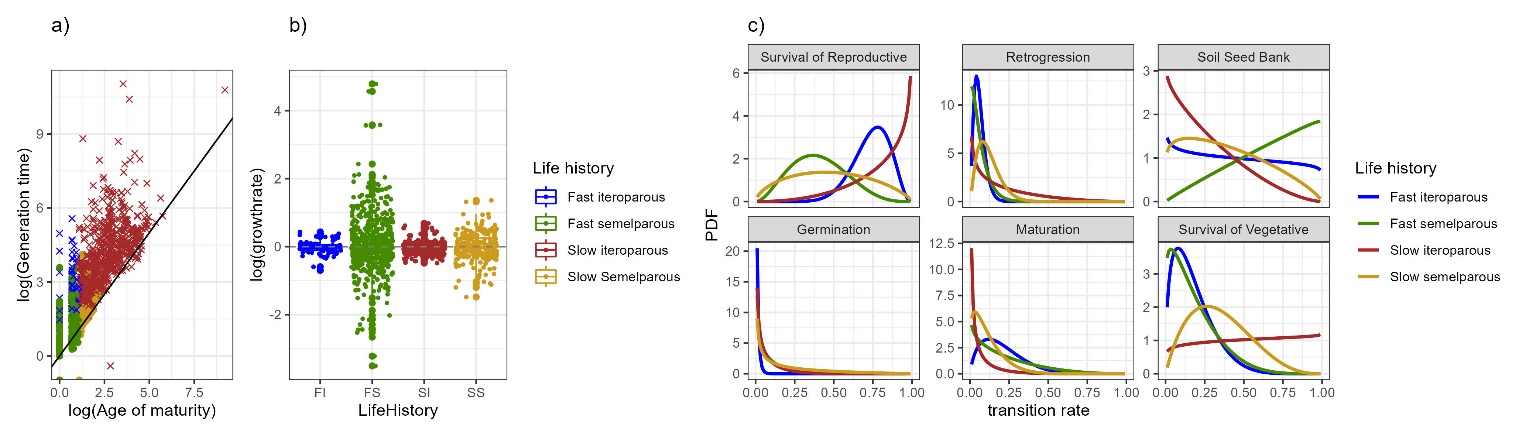

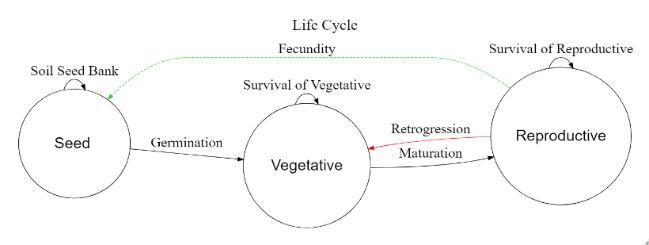


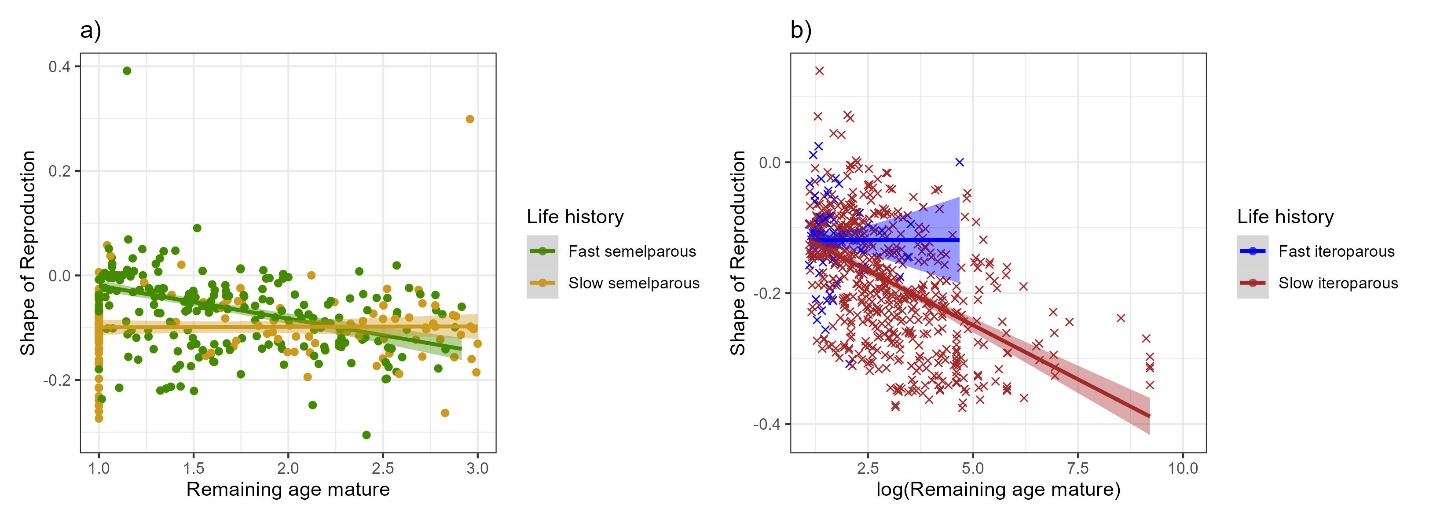
Figure S6: The relationship of shape of reproduction (Baudisch & Stott, 2019b) to a) the adult longevity, $\alpha_{age}$ remaining age after reaching reproductive maturity, for semelparous populations and b) the log-transformed adult longevity $\alpha_{age}$ for iteroparous populations. Mode of reproduction estimates are from 1,606 stage-based population matrices representing 317 species from the COMPADRE plants matrix database (COMPADRE, 2022). Each matrix is classified as itero- or semelparous and ‘fast’ or ‘slow’.

Table S1: Number of species within each plant family included in the simulations.

| Family | Number of species |
| --- | --- |
| Acanthaceae | 1 |
| Adoxaceae | 1 |
| Annonaceae | 1 |
| Araceae | 1 |
| Araliaceae | 1 |
| Aspleniaceae | 1 |
| Betulaceae | 1 |
| Boraginaceae | 1 |
| Colchicaceae | 1 |
| Convolvulaceae | 1 |
| Crassulaceae | 1 |
| Euphorbiaceae | 1 |
| Fzcaceae | 1 |
| Geraniaceae | 1 |
| Hypericaceae | 1 |
| Iridaceae | 1 |
| Juglandaceae | 1 |
| Laminariaceae | 1 |
| Lecythidaceae | 1 |
| Liliaceae | 1 |
| Linaceae | 1 |
| Marantaceae | 1 |
| Nothofagaceae | 1 |
| Onagraceae | 1 |
| Oxalidaceae | 1 |
| Paeoniaceae | 1 |
| Papaveraceae | 1 |
| Parmeliaceae | 1 |
| Podocarpaceae | 1 |
| Santalaceae | 1 |
| Sapotaceae | 1 |
| Saxifragaceae | 1 |
| Simaroubaceae | 1 |
| Solanaceae | 1 |
| Thymelaeaceae | 1 |
| Vochysiaceae | 1 |
| Zygophyllaceae | 1 |
| Amaranthaceae | 2 |
| Amaryllidaceae | 2 |
| Asparagaceae | 2 |
| Burseraceae | 2 |
| Caprifoliaceae | 2 |
| Cupressaceae | 2 |
| Dipterocarpaceae | 2 |
| Ericaceae | 2 |
| Fagaceae | 2 |
| Gesneriaceae | 2 |
| Malvaceae | 2 |
| Phrymaceae | 2 |
| Phyllanthaceae | 2 |
| Polemoniaceae | 2 |
| Polygonaceae | 2 |
| Proteaceae | 2 |
| Rosaceae | 2 |
| Scrophulariaceae | 2 |
| Lamiaceae | 3 |
| Lauraceae | 3 |
| Magnoliaceae | 3 |
| Meliaceae | 3 |
| Plantaginaceae | 3 |
| Rubiaceae | 3 |
| Apiaceae | 4 |
| Melanthiaceae | 4 |
| Zamiaceae | 4 |
| Myrtaceae | 5 |
| Plumbaginaceae | 5 |
| Primulaceae | 5 |
| Brassicaceae | 6 |
| Caryophyllaceae | 6 |
| Cistaceae | 6 |
| Pinaceae | 6 |
| Ranunculaceae | 6 |
| Poaceae | 7 |
| Bromeliaceae | 9 |
| Orchidaceae | 9 |
| Arecaceae | 17 |
| Fabaceae | 22 |
| Asteraceae | 23 |
| Cactaceae | 27 |

Table S2: Mixed effects binomial regression of the odds of extinction within 10 and 100 simulated years for existing populations initiated at stable stage distriubtion for initial population sizes of [1, 10, 100, 500, or 1000] as explained by parity, pace, initial population size, simulated stochasticity, and their interactions. Parameter estimates and standard deviation are presented on the logit scale and represent the difference from the mean value. Initial population size was scaled and centered. Model fit using JAGS, 3 chains each with 10000 iterations (first 5,000 discarded), thinning to every sixth. Non-zero effects are highlighted. Note the change in non-zero effect of environmental stochasticity on initial population size between 10 and 100 years.

|  | Odds of extinction within 10 yrs | | | |  | Odds of extinction within 100 yrs | | | |
| --- | --- | --- | --- | --- | --- | --- | --- | --- | --- |
|  | Mean | Std. Dev. | Lower 95% HPDI | Upper 95% HPDI |  | Mean | Std. Dev. | Lower 95% HPDI | Upper 95% HPDI |
| (Intercept) | -0.63 | 0.01 | -0.65 | -0.62 |  | 1.47 | 0.01 | 1.45 | 1.48 |
| Pace [Fast] | 0.08 | 4.68 | -9.04 | 9.34 |  | 0.11 | 4.71 | -8.97 | 9.34 |
| Pace [Slow] | -0.08 | 4.68 | -9.34 | 9.04 |  | -0.11 | 4.71 | -9.34 | 8.97 |
| Parity [Iteroparous] | -0.05 | 4.64 | -9.47 | 9.05 |  | 0.00 | 4.59 | -9.12 | 8.69 |
| Parity [Semelparous] | 0.05 | 4.64 | -9.05 | 9.47 |  | 0.00 | 4.59 | -8.69 | 9.12 |
| Parity [Iteroparous] X Pace [Fast] | -0.09 | 5.95 | -12.16 | 11.76 |  | -0.13 | 5.99 | -12.02 | 11.57 |
| Parity [Iteroparous] X Pace [Slow] | -0.28 | 5.95 | -12.04 | 11.56 |  | -0.01 | 5.97 | -11.44 | 11.72 |
| Parity [Semelparous] X Pace [Fast] | 0.19 | 5.95 | -11.63 | 11.96 |  | -0.16 | 5.97 | -11.90 | 11.28 |
| Parity [Semelparous] X Pace [Slow] | 0.18 | 5.95 | -11.66 | 12.22 |  | 0.29 | 5.99 | -11.39 | 12.19 |
| Pace [Fast] X Initial Population Size | 0.27 | 0.01 | 0.25 | 0.30 |  | 0.09 | 0.01 | 0.08 | 0.11 |
| Pace [Slow] X Initial Population Size | -0.27 | 0.01 | -0.30 | -0.25 |  | -0.09 | 0.01 | -0.11 | -0.08 |
| Pace [Fast] X Asymptotic | 0.07 | 6.24 | -12.19 | 12.45 |  | 0.02 | 6.16 | -12.01 | 11.78 |
| Pace [Slow] X Asymptotic | 0.17 | 6.17 | -11.78 | 12.18 |  | 0.20 | 6.15 | -11.52 | 12.30 |
| Pace [Fast] X Demographic | -0.08 | 6.25 | -12.79 | 11.95 |  | -0.19 | 6.19 | -12.18 | 11.81 |
| Pace [Slow] X Demographic | -0.33 | 6.14 | -12.37 | 11.87 |  | -0.26 | 6.24 | -12.73 | 12.16 |
| Pace [Fast] X Environmental | 0.04 | 6.26 | -12.40 | 11.87 |  | 0.18 | 6.30 | -11.92 | 12.77 |
| Pace [Slow] X Environmental | 0.24 | 6.29 | -11.74 | 12.87 |  | 0.26 | 6.24 | -12.00 | 12.45 |
| Pace [Fast] X Environ & Demo | -0.05 | 6.21 | -12.15 | 11.89 |  | -0.26 | 6.19 | -12.11 | 11.82 |
| Pace [Slow] X Environ & Demo | -0.05 | 6.36 | -12.35 | 12.78 |  | 0.04 | 6.21 | -11.94 | 12.09 |
| Parity [Iteroparous] X Initial Population Size | -0.33 | 0.01 | -0.35 | -0.31 |  | -0.21 | 0.01 | -0.23 | -0.20 |
| Parity [Semelparous] X Initial Population Size | 0.33 | 0.01 | 0.31 | 0.35 |  | 0.21 | 0.01 | 0.20 | 0.23 |
| Parity [Iteroparous] X Asymptotic | 0.06 | 6.23 | -11.86 | 12.98 |  | 0.35 | 6.30 | -12.02 | 12.68 |
| Parity [Semelparous] X Asymptotic | 0.20 | 6.33 | -12.50 | 12.52 |  | -0.08 | 6.29 | -12.35 | 12.17 |
| Parity [Iteroparous] X Demographic | -0.33 | 6.30 | -12.87 | 12.20 |  | -0.28 | 6.17 | -12.74 | 11.74 |
| Parity [Semelparous] X Demographic | 0.22 | 6.19 | -12.01 | 12.47 |  | 0.20 | 6.35 | -12.42 | 13.05 |
| Parity [Iteroparous] X Environmental | 0.19 | 6.18 | -11.89 | 11.57 |  | 0.72 | 6.21 | -11.33 | 13.01 |
| Parity [Semelparous] X Environmental | 0.10 | 6.14 | -12.06 | 12.15 |  | -0.57 | 6.20 | -12.74 | 11.49 |
| Parity [Iteroparous] X Environ & Demo | -0.48 | 6.19 | -12.59 | 11.38 |  | -0.53 | 6.28 | -12.78 | 11.81 |
| Parity [Semelparous] X Environ & Demo | 0.04 | 6.23 | -12.45 | 12.16 |  | 0.19 | 6.11 | -11.71 | 12.11 |
| Initial Population Size | -1.84 | 0.01 | -1.86 | -1.82 |  | -0.71 | 0.01 | -0.72 | -0.69 |
| Initial Population Size X Asymptotic | 0.04 | 0.02 | 0.01 | 0.07 |  | 0.06 | 0.01 | 0.04 | 0.08 |
| Initial Population Size X Demographic | 0.07 | 0.02 | 0.03 | 0.10 |  | 0.15 | 0.01 | 0.13 | 0.17 |
| Initial Population Size X Environmental | 0.22 | 0.01 | 0.19 | 0.25 |  | -0.13 | 0.01 | -0.15 | -0.10 |
| Initial Population Size X Environ & Demo | -0.33 | 0.02 | -0.37 | -0.29 |  | -0.09 | 0.01 | -0.11 | -0.07 |
| Asymptotic | 0.13 | 6.15 | -12.05 | 12.52 |  | -0.15 | 6.12 | -11.96 | 12.13 |
| Demographic Stochasticity | -0.15 | 6.25 | -12.14 | 12.07 |  | -0.22 | 6.11 | -12.17 | 11.66 |
| Environmental Stochasticity | 0.35 | 6.15 | -11.43 | 12.24 |  | 0.55 | 6.07 | -11.13 | 12.61 |
| Environmental and Demographic Stochasticity | -0.34 | 6.07 | -12.33 | 11.76 |  | -0.18 | 6.05 | -12.01 | 12.20 |
| deviance | 191258.80 | 6.16 | 191248.79 | 191272.58 |  | 185283.42 | 6.14 | 185273.32 | 185296.56 |

Table S3: Mixed effects binomial regression of the odds of extinction within 10 and 100 simulated years for exisiting populations initiated at stable stage distriubtion for initial population sizes of [1, 10, 100, 500, or 1000] as explained by shape of reproduction, pace, initial population size, simulated stochasticity, and their interactions. Parameter estimates and standard deviation are presented on the logit scale and represent the difference from the mean value. Initial population size was scaled and centered. Model fit using JAGS, 3 chains each with 10000 iterations (first 5,000 discarded), thinning to every sixth. Non-zero effects are highlighted in grey. Differences in non-zero effects highlighted in red.

|  | Extinction risk within 10 years | | | |  | Extinction risk within 100 years | | | |
| --- | --- | --- | --- | --- | --- | --- | --- | --- | --- |
|  | Mean | Std. Dev. | Lower 95% HPDI | Upper 95% HPDI |  | Mean | Std. Dev. | Lower 95% HPDI | Upper 95% HPDI |
| (Intercept) | -0.45 | 0.01 | -0.47 | -0.44 |  | 1.82 | 0.01 | 1.80 | 1.84 |
| Pace [Fast] | 0.44 | 3.14 | -5.81 | 6.78 |  | 0.26 | 3.19 | -6.07 | 6.54 |
| Pace [Slow] | -0.44 | 3.14 | -6.78 | 5.81 |  | -0.26 | 3.19 | -6.54 | 6.07 |
| Shape of Reproduction X Pace [Fast] | -0.23 | 0.01 | -0.25 | -0.21 |  | -0.42 | 0.01 | -0.44 | -0.40 |
| Shape of Reproduction X Pace [Slow] | 0.23 | 0.01 | 0.21 | 0.25 |  | 0.42 | 0.01 | 0.40 | 0.44 |
| Pace [Fast] X Population Size | 0.43 | 0.01 | 0.41 | 0.45 |  | 0.08 | 0.01 | 0.07 | 0.09 |
| Pace [Slow] X Population Size | -0.43 | 0.01 | -0.45 | -0.41 |  | -0.08 | 0.01 | -0.09 | -0.07 |
| Pace [Fast] X Asymptotic | -0.03 | 5.77 | -11.51 | 11.27 |  | -0.01 | 5.94 | -11.45 | 11.80 |
| Pace [Slow] X Asymptotic | 0.03 | 6.04 | -11.72 | 11.84 |  | 0.07 | 5.83 | -11.09 | 11.23 |
| Pace [Fast] X Demographic | -0.10 | 5.98 | -11.80 | 11.54 |  | -0.15 | 6.04 | -11.81 | 11.60 |
| Pace [Slow] X Demographic | -0.43 | 5.90 | -11.87 | 11.62 |  | -0.29 | 6.00 | -12.24 | 11.21 |
| Pace [Fast] X Environmental | 0.43 | 6.01 | -11.22 | 12.14 |  | 0.47 | 5.85 | -10.87 | 12.02 |
| Pace [Slow] X Environmental | 0.42 | 5.87 | -11.18 | 11.64 |  | 0.14 | 6.02 | -11.41 | 12.11 |
| Pace [Fast] X Environ & Demo | -0.03 | 5.89 | -11.38 | 11.67 |  | -0.07 | 6.04 | -11.48 | 12.05 |
| Pace [Slow] X Environ & Demo | -0.30 | 5.84 | -11.39 | 11.03 |  | -0.17 | 6.02 | -11.92 | 11.80 |
| Shape of Reproduction | -0.20 | 0.01 | -0.21 | -0.18 |  | -0.53 | 0.01 | -0.55 | -0.51 |
| Shape of Reproduction X Asymptotic | 0.01 | 0.01 | -0.02 | 0.03 |  | -0.06 | 0.01 | -0.09 | -0.04 |
| Shape of Reproduction X Demographic | 0.16 | 0.01 | 0.14 | 0.18 |  | 0.43 | 0.01 | 0.41 | 0.46 |
| Shape of Reproduction X Environmental | -0.22 | 0.01 | -0.24 | -0.19 |  | -0.74 | 0.02 | -0.78 | -0.71 |
| Shape of Reproduction X Environ & Demo | 0.05 | 0.01 | 0.03 | 0.08 |  | 0.37 | 0.01 | 0.35 | 0.40 |
| Population size | -1.79 | 0.01 | -1.81 | -1.77 |  | -0.79 | 0.01 | -0.80 | -0.78 |
| Population size X Shape of Reproduction | 0.06 | 0.01 | 0.04 | 0.08 |  | 0.24 | 0.01 | 0.22 | 0.25 |
| Population size X Asymptotic | 0.02 | 0.02 | -0.01 | 0.05 |  | 0.06 | 0.01 | 0.04 | 0.08 |
| Population size X Demographic | 0.09 | 0.02 | 0.05 | 0.12 |  | 0.15 | 0.01 | 0.14 | 0.17 |
| Population size X Environmental | 0.19 | 0.01 | 0.16 | 0.22 |  | -0.16 | 0.01 | -0.18 | -0.14 |
| Population size X Environ & Demo | -0.30 | 0.02 | -0.34 | -0.26 |  | -0.06 | 0.01 | -0.07 | -0.04 |
| Asymptotic | 0.35 | 5.00 | -9.29 | 10.23 |  | 0.07 | 4.94 | -9.59 | 9.52 |
| Demographic Stochasticity | -0.19 | 5.04 | -9.97 | 9.33 |  | -0.43 | 5.10 | -10.54 | 9.13 |
| Environmental Stochasticity | 0.25 | 5.05 | -9.47 | 9.72 |  | 0.80 | 5.00 | -8.82 | 10.66 |
| Environmental and Demographic Stochasticity | -0.42 | 4.95 | -10.19 | 9.01 |  | -0.45 | 5.11 | -10.71 | 9.31 |
| deviance | 191978.68 | 6.23 | 191968.74 | 191993.10 |  | 182598.77 | 6.09 | 182588.60 | 182611.93 |

Table S4: Mixed effects binomial regression of the odds of extinction within 10 and 100 simulated years for novel populations initiated as either seed or seedlings (the first non-dormant stage) for initial population sizes of [1, 10, 100, 500, or 1000] as explained by parity, pace, initial population size, simulated stochasticity, and their interactions. Parameter estimates and standard deviation are presented on the logit scale and represent the difference from the mean value. Initial population size was scaled and centered. Model fit using JAGS, 3 chains each with 10000 iterations (first 5,000 discarded), thinning to every sixth. Non-zero effects are highlighted in grey. Differences in non-zero effects highlighted in red.

| **Categorical Parity - Duration** | Extinction risk within 10 years | | | |  | Extinction risk within 100 years | | | |
| --- | --- | --- | --- | --- | --- | --- | --- | --- | --- |
|  | **Mean** | **SD** | **Lower HPDI 2.50%** | **Upper HPDI 97.50%** |  | **Mean** | **SD** | **Lower HPDI 2.50%** | **Upper HPDI 97.50%** |
| (Intercept) | -0.06 | 0.01 | -0.08 | -0.05 |  | 1.69 | 0.01 | 1.67 | 1.71 |
| Pace [Fast] | -0.10 | 5.30 | -10.28 | 10.34 |  | -0.08 | 5.17 | -10.30 | 10.08 |
| Pace [Slow] | 0.10 | 5.30 | -10.34 | 10.28 |  | 0.08 | 5.17 | -10.08 | 10.30 |
| Parity [Iteroparous] | 0.05 | 5.29 | -10.25 | 9.99 |  | 0.38 | 5.36 | -10.68 | 10.91 |
| Parity [Semelparous] | -0.05 | 5.29 | -9.99 | 10.25 |  | -0.38 | 5.36 | -10.91 | 10.68 |
| Initial population size | -1.36 | 0.01 | -1.37 | -1.35 |  | -0.71 | 0.01 | -0.72 | -0.70 |
| Founding stage [Seed] | 0.29 | 5.23 | -9.92 | 10.53 |  | 0.51 | 5.36 | -10.09 | 10.68 |
| Founding stage [Seedling] | -0.29 | 5.23 | -10.53 | 9.92 |  | -0.51 | 5.36 | -10.68 | 10.09 |
| Asymptotic | -0.07 | 6.55 | -13.14 | 12.39 |  | 0.18 | 6.76 | -13.12 | 13.43 |
| Demographic Stochasticity | -0.26 | 6.86 | -13.53 | 13.44 |  | -0.25 | 6.82 | -13.67 | 12.93 |
| Environmental Stochasticity | 0.46 | 6.76 | -12.72 | 13.07 |  | 0.41 | 6.59 | -12.41 | 13.13 |
| Environmental and Demographic Stochasticity | -0.13 | 6.63 | -13.22 | 12.63 |  | -0.33 | 6.79 | -13.37 | 13.01 |
| Pace [Fast] × Parity [Iteroparous] | -0.29 | 6.27 | -12.38 | 12.12 |  | -0.12 | 6.21 | -12.36 | 12.36 |
| Pace [Slow] × Parity [Iteroparous] | 0.76 | 6.21 | -11.47 | 12.49 |  | 0.84 | 6.16 | -11.54 | 13.21 |
| Pace [Fast] × Parity [Semelparous] | -0.16 | 6.21 | -11.89 | 12.06 |  | -0.11 | 6.16 | -12.49 | 12.27 |
| Pace [Slow] × Parity [Semelparous] | -0.31 | 6.27 | -12.71 | 11.79 |  | -0.61 | 6.21 | -13.08 | 11.64 |
| Pace [Fast] × Initial population size | -0.04 | 0.01 | -0.05 | -0.03 |  | -0.02 | 0.01 | -0.03 | -0.01 |
| Pace [Slow] × Initial population size | 0.04 | 0.01 | 0.03 | 0.05 |  | 0.02 | 0.01 | 0.01 | 0.03 |
| Pace [Fast] × Founding stage [Seed] | 0.23 | 6.18 | -11.62 | 12.35 |  | 0.34 | 6.17 | -12.01 | 11.86 |
| Pace [Slow] × Founding stage [Seed] | 0.21 | 6.25 | -11.93 | 12.65 |  | 0.02 | 6.20 | -12.03 | 12.28 |
| Pace [Fast] × Founding stage [Seedling] | -0.34 | 6.25 | -12.78 | 11.81 |  | -0.34 | 6.20 | -12.62 | 11.72 |
| Pace [Slow] × Founding stage [Seedling] | -0.11 | 6.18 | -12.21 | 11.74 |  | -0.02 | 6.17 | -11.54 | 12.33 |
| Asymptotic × Pace [Fast] | 0.11 | 6.42 | -12.51 | 12.89 |  | 0.00 | 6.62 | -13.05 | 12.90 |
| Asymptotic × Pace [Slow] | 0.20 | 6.39 | -12.83 | 12.41 |  | 0.07 | 6.43 | -13.14 | 12.45 |
| Demographic × Pace [Fast] | -0.25 | 6.35 | -12.67 | 12.37 |  | -0.25 | 6.36 | -12.60 | 12.80 |
| Demographic × Pace [Slow] | -0.12 | 6.36 | -12.53 | 12.23 |  | -0.07 | 6.37 | -12.64 | 12.36 |
| Environmental × Pace [Fast] | 0.20 | 6.59 | -13.18 | 12.80 |  | 0.42 | 6.42 | -12.29 | 13.19 |
| Environmental × Pace [Slow] | 0.08 | 6.29 | -12.25 | 12.22 |  | 0.38 | 6.48 | -11.92 | 12.73 |
| Environ & Demo × Pace [Fast] | -0.26 | 6.38 | -12.77 | 12.16 |  | -0.36 | 6.36 | -12.73 | 11.85 |
| Environ & Demo × Pace [Slow] | 0.04 | 6.70 | -12.85 | 13.25 |  | -0.19 | 6.48 | -13.42 | 12.42 |
| Parity [Iteroparous] × Founding stage [Seed] | 0.63 | 6.06 | -11.11 | 12.75 |  | 0.62 | 6.18 | -11.29 | 12.30 |
| Parity [Semelparous] × Founding stage [Seed] | -0.23 | 6.34 | -12.29 | 12.76 |  | -0.40 | 6.24 | -12.33 | 11.76 |
| Parity [Iteroparous] × Founding stage [Seedling] | -0.27 | 6.34 | -13.27 | 11.77 |  | -0.34 | 6.24 | -12.49 | 11.60 |
| Parity [Semelparous] × Founding stage [Seedling] | -0.13 | 6.06 | -12.27 | 11.61 |  | 0.12 | 6.18 | -11.59 | 12.04 |
| Parity [Iteroparous] × Initial population size | 0.04 | 0.01 | 0.03 | 0.06 |  | -0.09 | 0.01 | -0.10 | -0.07 |
| Parity [Semelparous] × Initial population size | -0.04 | 0.01 | -0.06 | -0.03 |  | 0.09 | 0.01 | 0.07 | 0.10 |
| Asymptotic × Parity [Iteroparous] | 0.11 | 6.47 | -12.03 | 12.56 |  | 0.21 | 6.41 | -12.81 | 12.37 |
| Asymptotic × Parity [Semelparous] | -0.05 | 6.47 | -12.61 | 12.98 |  | -0.34 | 6.53 | -12.89 | 12.46 |
| Demographic × Parity [Iteroparous] | -0.16 | 6.47 | -12.80 | 12.20 |  | -0.37 | 6.49 | -12.75 | 12.44 |
| Demographic × Parity [Semelparous] | 0.21 | 6.53 | -12.35 | 12.63 |  | 0.13 | 6.34 | -12.71 | 12.48 |
| Environmental × Parity [Iteroparous] | 0.26 | 6.37 | -11.82 | 13.14 |  | 0.79 | 6.62 | -11.90 | 13.79 |
| Environmental × Parity [Semelparous] | -0.04 | 6.41 | -12.70 | 12.70 |  | -0.52 | 6.47 | -12.83 | 12.20 |
| Environ & Demo × Parity [Iteroparous] | -0.10 | 6.38 | -12.80 | 12.26 |  | 0.25 | 6.35 | -12.38 | 12.93 |
| Environ & Demo × Parity [Semelparous] | -0.23 | 6.28 | -12.32 | 12.18 |  | -0.15 | 6.52 | -13.22 | 12.83 |
| Founding stage [Seed] × Initial population size | 0.27 | 0.01 | 0.26 | 0.28 |  | -0.11 | 0.00 | -0.12 | -0.10 |
| Founding stage [Seedling] × Initial population size | -0.27 | 0.01 | -0.28 | -0.26 |  | 0.11 | 0.00 | 0.10 | 0.12 |
| Asymptotic × Initial population size | -0.06 | 0.01 | -0.08 | -0.04 |  | 0.05 | 0.01 | 0.03 | 0.06 |
| Demographic × Initial population size | 0.08 | 0.01 | 0.06 | 0.10 |  | 0.14 | 0.01 | 0.13 | 0.15 |
| Environmental × Initial population size | 0.25 | 0.01 | 0.23 | 0.26 |  | -0.07 | 0.01 | -0.08 | -0.05 |
| Environ & Demo × Initial population size | -0.27 | 0.01 | -0.29 | -0.25 |  | -0.12 | 0.01 | -0.13 | -0.11 |
| Asymptotic × Founding stage [Seed] | 0.13 | 6.25 | -12.19 | 12.81 |  | -0.16 | 6.34 | -12.74 | 12.22 |
| Asymptotic × Founding stage [Seedling] | -0.14 | 6.47 | -12.93 | 12.15 |  | 0.02 | 6.37 | -12.66 | 12.35 |
| Demographic × Founding stage [Seed] | -0.09 | 6.49 | -12.52 | 12.55 |  | -0.41 | 6.33 | -12.20 | 12.12 |
| Demographic × Founding stage [Seedling] | -0.18 | 6.49 | -13.06 | 12.29 |  | 0.06 | 6.49 | -12.52 | 12.62 |
| Environmental × Founding stage [Seed] | 0.77 | 6.55 | -12.21 | 14.00 |  | 0.32 | 6.30 | -12.22 | 12.58 |
| Environmental × Founding stage [Seedling] | -0.05 | 6.41 | -12.46 | 12.42 |  | -0.01 | 6.38 | -12.11 | 12.60 |
| Environ & Demo × Founding stage [Seed] | 0.01 | 6.56 | -12.90 | 12.99 |  | 0.03 | 6.49 | -12.96 | 12.13 |
| Environ & Demo × Founding stage [Seedling] | -0.46 | 6.48 | -13.12 | 12.20 |  | 0.15 | 6.20 | -12.22 | 12.48 |
| deviance | 377434.62 | 7.20 | 377422.88 | 377450.67 |  | 352595.38 | 7.18 | 352583.37 | 352611.16 |

Table S5: Mixed effects binomial regression of the odds of extinction within 10 and 100 simulated years for novel populations initiated as either seed or seedlings (the first non-dormant stage) for initial population sizes of [1, 10, 100, 500, or 1000] as explained by shape of reproduction, pace, initial population size, simulated stochasticity, and their interactions. Parameter estimates and standard deviation are presented on the logit scale and represent the difference from the mean value. Initial population size was scaled and centered. Model fit using JAGS, 3 chains each with 10000 iterations (first 5,000 discarded), thinning to every sixth. Non-zero effects are highlighted in grey. Differences in non-zero effects highlighted in red.

| **Continuous Shape - Intensity** | Extinction risk within 10 years | | | |  | Extinction risk within 100 years | | | |
| --- | --- | --- | --- | --- | --- | --- | --- | --- | --- |
|  | **Mean** | **SD** | **Lower HPDI 2.50%** | **Upper HPDI 97.50%** |  | **Mean** | **SD** | **Lower HPDI 2.50%** | **Upper HPDI 97.50%** |
| (Intercept) | 0.17 | 0.01 | 0.15 | 0.19 |  | 2.59 | 0.02 | 2.55 | 2.63 |
| Pace [Fast] | -0.27 | 4.74 | -9.79 | 8.99 |  | -0.37 | 4.67 | -9.72 | 8.79 |
| Pace [Slow] | 0.27 | 4.74 | -8.99 | 9.79 |  | 0.37 | 4.67 | -8.79 | 9.72 |
| Shape of Reproduction | -0.33 | 0.01 | -0.34 | -0.31 |  | -0.62 | 0.01 | -0.65 | -0.60 |
| Initial population size | -1.66 | 0.01 | -1.68 | -1.64 |  | -0.78 | 0.01 | -0.80 | -0.76 |
| Founding stage [Seed] | 0.74 | 4.67 | -8.65 | 9.74 |  | 0.50 | 4.71 | -8.81 | 9.60 |
| Founding stage [Seedling] | -0.74 | 4.67 | -9.74 | 8.65 |  | -0.50 | 4.71 | -9.60 | 8.81 |
| Asymptotic | 0.34 | 6.07 | -11.35 | 12.00 |  | 0.11 | 6.08 | -11.95 | 11.91 |
| Demographic Stochasticity | -0.41 | 6.09 | -12.77 | 11.28 |  | -0.57 | 6.28 | -13.27 | 11.62 |
| Environmental Stochasticity | 0.59 | 6.17 | -11.11 | 12.69 |  | 0.93 | 6.15 | -11.22 | 12.94 |
| Environmental and Demographic Stochasticity | -0.53 | 6.21 | -12.68 | 11.81 |  | -0.47 | 6.05 | -12.38 | 11.26 |
| Pace [Fast] × Shape of Reproduction | -0.05 | 0.01 | -0.06 | -0.03 |  | 0.68 | 0.01 | 0.67 | 0.70 |
| Pace [Slow] × Shape of Reproduction | 0.05 | 0.01 | 0.03 | 0.06 |  | -0.68 | 0.01 | -0.70 | -0.67 |
| Pace [Fast] × Initial population size | 0.04 | 0.01 | 0.02 | 0.06 |  | 0.02 | 0.01 | 0.01 | 0.04 |
| Pace [Slow] × Initial population size | -0.04 | 0.01 | -0.06 | -0.02 |  | -0.02 | 0.01 | -0.04 | -0.01 |
| Pace [Fast] × Founding stage [Seed] | 0.18 | 5.88 | -11.32 | 11.98 |  | 0.47 | 5.88 | -10.99 | 12.19 |
| Pace [Slow] × Founding stage [Seed] | 0.46 | 6.05 | -11.18 | 11.97 |  | 0.55 | 6.16 | -11.28 | 12.28 |
| Pace [Fast] × Founding stage [Seedling] | -0.20 | 6.05 | -11.70 | 11.45 |  | -0.69 | 6.16 | -12.43 | 11.11 |
| Pace [Slow] × Founding stage [Seedling] | -0.44 | 5.88 | -12.23 | 11.08 |  | -0.32 | 5.88 | -12.04 | 11.16 |
| Asymptotic × Pace [Fast] | -0.07 | 6.16 | -12.28 | 11.95 |  | 0.01 | 6.25 | -12.40 | 12.72 |
| Asymptotic × Pace [Slow] | 0.04 | 6.22 | -12.28 | 12.32 |  | -0.07 | 6.15 | -12.09 | 12.34 |
| Demographic × Pace [Fast] | 0.11 | 6.19 | -12.10 | 12.28 |  | -0.03 | 6.38 | -12.30 | 12.06 |
| Demographic × Pace [Slow] | 0.05 | 6.30 | -12.48 | 12.63 |  | -0.39 | 6.39 | -12.90 | 11.74 |
| Environmental × Pace [Fast] | 0.27 | 6.32 | -11.93 | 12.83 |  | 0.46 | 6.26 | -11.44 | 12.54 |
| Environmental × Pace [Slow] | 0.15 | 6.16 | -12.15 | 11.82 |  | 0.38 | 6.25 | -11.87 | 12.92 |
| Environ & Demo × Pace [Fast] | -0.37 | 6.18 | -12.03 | 11.76 |  | -0.30 | 6.25 | -12.35 | 12.22 |
| Environ & Demo × Pace [Slow] | -0.19 | 6.16 | -11.99 | 12.27 |  | -0.07 | 6.35 | -12.60 | 12.28 |
| Shape of Reproduction × Founding stage [Seed] | -0.21 | 0.01 | -0.23 | -0.20 |  | -0.08 | 0.01 | -0.11 | -0.06 |
| Shape of Reproduction × Founding stage [Seedling] | 0.21 | 0.01 | 0.20 | 0.23 |  | 0.08 | 0.01 | 0.06 | 0.11 |
| Initial population size × Shape of Reproduction | 0.08 | 0.01 | 0.06 | 0.10 |  | 0.08 | 0.01 | 0.06 | 0.10 |
| Asymptotic × Shape of Reproduction | 0.01 | 0.01 | -0.01 | 0.03 |  | -0.05 | 0.02 | -0.08 | -0.01 |
| Demographic × Shape of Reproduction | 0.08 | 0.01 | 0.06 | 0.10 |  | 0.31 | 0.02 | 0.28 | 0.34 |
| Environmental × Shape of Reproduction | 0.08 | 0.01 | 0.05 | 0.11 |  | -0.58 | 0.03 | -0.63 | -0.52 |
| Environ & Demo × Shape of Reproduction | -0.17 | 0.02 | -0.20 | -0.14 |  | 0.31 | 0.02 | 0.27 | 0.35 |
| Founding stage [Seed] × Initial population size | 0.57 | 0.01 | 0.55 | 0.59 |  | -0.08 | 0.01 | -0.10 | -0.07 |
| Founding stage [Seedling] × Initial population size | -0.57 | 0.01 | -0.59 | -0.55 |  | 0.08 | 0.01 | 0.07 | 0.10 |
| Asymptotic × Initial population size | -0.04 | 0.01 | -0.07 | -0.02 |  | 0.01 | 0.01 | -0.02 | 0.03 |
| Demographic × Initial population size | 0.11 | 0.01 | 0.09 | 0.14 |  | 0.15 | 0.01 | 0.12 | 0.17 |
| Environmental × Initial population size | 0.15 | 0.01 | 0.12 | 0.18 |  | -0.25 | 0.02 | -0.28 | -0.21 |
| Environ & Demo × Initial population size | -0.22 | 0.02 | -0.25 | -0.19 |  | 0.09 | 0.01 | 0.07 | 0.12 |
| Asymptotic × Founding stage [Seed] | -0.08 | 6.22 | -12.18 | 11.89 |  | -0.20 | 6.42 | -12.82 | 12.60 |
| Asymptotic × Founding stage [Seedling] | -0.13 | 6.14 | -12.51 | 11.55 |  | 0.12 | 6.08 | -11.51 | 12.05 |
| Demographic × Founding stage [Seed] | -0.32 | 6.16 | -12.41 | 11.80 |  | -0.71 | 6.37 | -12.83 | 11.65 |
| Demographic × Founding stage [Seedling] | -0.22 | 6.18 | -12.16 | 12.09 |  | 0.09 | 6.38 | -12.29 | 13.11 |
| Environmental × Founding stage [Seed] | 0.66 | 6.16 | -11.23 | 12.91 |  | 1.46 | 6.25 | -11.12 | 13.63 |
| Environmental × Founding stage [Seedling] | -0.02 | 6.24 | -12.31 | 11.67 |  | -0.29 | 6.35 | -12.83 | 12.04 |
| Environ & Demo × Founding stage [Seed] | 0.29 | 6.26 | -11.22 | 12.85 |  | -0.35 | 6.08 | -12.32 | 11.72 |
| Environ & Demo × Founding stage [Seedling] | -0.18 | 6.23 | -11.92 | 11.73 |  | -0.12 | 6.31 | -12.73 | 12.38 |
| deviance | ###### | 7.18 | 178338.91 | 178367.02 |  | 116600.15 | 7.07 | 116588.69 | 116615.92 |

References:

Baudisch, A., & Stott, I. (2019a). A pace and shape perspective on fertility. *Methods in Ecology and Evolution*, *10*(11), 1941–1951. https://doi.org/10.1111/2041-210x.13289

Baudisch, A., & Stott, I. (2019b). A pace and shape perspective on fertility. *METHODS IN ECOLOGY AND EVOLUTION*, *10*(11), 1941–1951. https://doi.org/10.1111/2041-210X.13289

Caswell, H. (2001). *Matrix population models: construction, analysis, and interpretation*. http://catalog.hathitrust.org/api/volumes/oclc/44619483.html

Chamberlain, S. A., Hovick, S. M., Dibble Christopher J. and Rasmussen, N. L., van Allen, B. G., Maitner Brian S. and Ahern, J. R., Bell-Dereske, L. P., Roy Christopher L. and Meza-Lopez, M., Carrillo, J., Siemann, E., Lajeunesse, M. J., & Whitney, K. D. (2012). Does phylogeny matter? Assessing the impact of phylogenetic information in ecological meta-analysis. *ECOLOGY LETTERS*, *15*(6), 627–636. https://doi.org/10.1111/j.1461-0248.2012.01776.x

Chichorro, F., Juslen, A., & Cardoso, P. (2019). A review of the relation between species traits and extinction risk. *Biological Conservation*, *237*, 220–229. https://doi.org/10.1016/j.biocon.2019.07.001

Cochran, M. E., & Ellner, S. (1992). Simple methods for calculating age-based life-history parameters for stage-structured populaitons. *Ecological Monographs*, *62*(3), 345–364. https://doi.org/10.2307/2937115

COMPADRE. (2022, May 11). *COMPADRE Plants Matrix Database* (1.3). https://www.compadre-db.org

Felsenstein, J. (1985). Phylogenies and the comparative method. *The American Naturalist*, *125*, 1–15.

Guo, Y., Lu, W., Che, Z., Cao, J., Yang, H., & Huang, X. (2022). A meta-analysis on the relationship between seed size, seed shape and persistence in soil seed bank. *PAKISTAN JOURNAL OF BOTANY*, *54*(3), 925–930. https://doi.org/10.30848/PJB2022-3(29)

Janovsky, Z., Herben, T., & Klimesova, J. (2017). Accounting for clonality in comparative plant demography - growth or reproduction? *FOLIA GEOBOTANICA*, *52*(3–4, SI), 433–442. https://doi.org/10.1007/s12224-017-9301-4

Jones, O. R., Barks, P., Stott, I., James Tamora D. and Levin, S., Petry, W. K., Capdevila, P., Che-Castaldo, J., Jackson, J., Romer, G., Schuette, C., Thomas, C. C., & Salguero-Gomez, R. (2022). Rcompadre and Rage-Two R packages to facilitate the use of the COMPADRE and COMADRE databases and calculation of life-history traits from matrix population models. *METHODS IN ECOLOGY AND EVOLUTION*, *13*(4), 770–781. https://doi.org/10.1111/2041-210X.13792

Kembel, S. W., Cowan, P. D., Helmus, M. R., Cornwell, W. K., Morlon, H., Ackerly, D. D., Blomberg, S. P., & Webb, C. O. (2010). Picante: R tools for integrating phylogenies and ecology. *BIOINFORMATICS*, *26*(11), 1463–1464. https://doi.org/10.1093/bioinformatics/btq166

Koons, D. N., Iles, D. T., Schaub, M., & Caswell, H. (2016). A life-history perspective on the demographic drivers of structured population dynamics in changing environments. *ECOLOGY LETTERS*, *19*(9), 1023–1031. https://doi.org/10.1111/ele.12628

Qian, H., & Jin, Y. (2016). An updated megaphylogeny of plants, a tool for generating plant phylogenies and an analysis of phylogenetic community structure. *Journal of Plant Ecology*, *9*(2), 233–239. https://doi.org/10.1093/jpe/rtv047

Saatkamp, A., Poschlod, P., & Venable, D. L. (2014). The functional role of soil seed banks in natural communities. In R. S. Gallagher (Ed.), *Seeds: the ecology of regeneration in plant communities, 3rd edition* (pp. 263–295).

Salguero-Gómez, R. (2017). Applications of the fast–slow continuum and reproductive strategy framework of plant life histories. *New Phytologist*, *213*(4), 1618–1624. https://doi.org/10.1111/nph.14289

Schmid, M., Paniw, M., Postuma, M., Ozgul, A., & Guillaume, F. (2022). A Trade-Off between robustness to environmental fluctuations and speed of evolution. *AMERICAN NATURALIST*. https://doi.org/10.1086/719654

Vasconcelos, T. (2023). A trait-based approach to determining principles of plant biogeography. *American Journal of Botany*. https://doi.org/10.1002/ajb2.16127
